# Parenting interventions for parents of children with type 1 diabetes—a systematic review

**DOI:** 10.1093/jpepsy/jsaf078

**Published:** 2025-09-22

**Authors:** Mandy Jansen, Paul G Voorhoeve, Lianne Wiltink, Judith B Prins, Giesje Nefs

**Affiliations:** Department of Medical Psychology, Radboud University Medical Center, Nijmegen, The Netherlands; CWZ Academy, Canisius Wilhelmina Hospital, Nijmegen, The Netherlands; Center for Pediatric and Adolescent Diabetes, Vivendia, Nijmegen, The Netherlands; Center for Pediatric and Adolescent Diabetes, Vivendia, Nijmegen, The Netherlands; Department of Pediatrics, Canisius Wilhelmina Hospital, Nijmegen, The Netherlands; Center for Pediatric and Adolescent Diabetes, Vivendia, Nijmegen, The Netherlands; De Kleine Berg, Private practice, Nijmegen, The Netherlands; Department of Medical Psychology, Radboud University Medical Center, Nijmegen, The Netherlands; Department of Medical Psychology, Radboud University Medical Center, Nijmegen, The Netherlands; Center for Focused Diabetes Care and Research, Diabeter, Rotterdam, The Netherlands; Department of Medical and Clinical Psychology, Center of Research on Psychological Disorders and Somatic Diseases, Tilburg University, Tilburg, The Netherlands; Diabeter Centrum Amsterdam, Specialized Treatment Center, Amsterdam, The Netherlands

**Keywords:** diabetes, parent–adolescent communication, family functioning, parent psychosocial functioning, psychosocial intervention, systematic review

## Abstract

**Objectives:**

This systematic review (PROSPERO ID: CRD42022356654, AMNR junior research grant) evaluated the effectiveness of parenting interventions in pediatric type 1 diabetes, designed to enhance supportive parenting behaviors, in improving family dynamics, parent-, child-, and diabetes-related outcomes.

**Methods:**

We systematically searched PubMed, EMBASE, Cochrane, CINAHL, and PsycINFO for studies from 1980 to February 25, 2025. We included reports of controlled and uncontrolled studies describing quantitative change. Data were synthesized narratively, and intervention content was coded according to a behavioral taxonomy. Risk of bias was assessed using Cochrane’s Risk of Bias (2.0) tool and the ROBINS-I tool for controlled and uncontrolled studies, respectively.

**Results:**

After screening 12,654 reports, we included 51 studies (across 72 reports) describing findings of 37 unique interventions. Most studies and outcomes had an increased risk of bias. Whereas overall effects were mixed, intensive, targeted interventions had the most impact on psychosocial and diabetes outcomes. Some preventive interventions and, notably, control groups also showed effects, with most promising effects in subgroups. Many preventive intervention studies were underpowered. A diabetes-specific focus seemed necessary, although not sufficient, to affect diabetes outcomes. Several strategies were used to stimulate parents toward changing their own and—ultimately—their children’s behavior, although individual components could not be uniquely related to intervention effectiveness.

**Conclusions:**

Targeted and preventive parenting interventions serve as a potential, although not exclusive, approach to improve psychosocial and diabetes outcomes. Future research should elucidate which families benefit from parenting interventions compared to other educational or supporting interventions, thereby delineating their essential intervention components.

Pediatric type 1 diabetes (T1D) affects the entire family due to its demanding treatment. Daily diabetes-care behaviors—including blood glucose monitoring, insulin administration, and management of diet and activity—are required to manage blood glucose and prevent short-term (e.g., hypoglycemia, hyperglycemia) and long-term complications (neuropathy, retinopathy, nephropathy) (Redondo et al., 2021). Even though the American Diabetes Association (ADA) recommends an HbA1c of <7.0% (53 mmol/mol) for children and adolescents (American Diabetes Association Professional Practice Committee, 2025), fewer than 20% of young people meet this recommendation (Demeterco-Berggren et al., 2022). As children still lack the necessary cognitive, physical, and emotional skills to complete these tasks independently, parents play a crucial role in diabetes management (Markowitz et al., 2015).

As children develop, parental involvement in diabetes care changes accordingly. In early and middle childhood, parents typically assume primary responsibility for diabetes management tasks, with children increasingly assisting in diabetes tasks depending on their abilities and interest (Markowitz et al., 2015). When the child begins to negotiate and assume more responsibilities, direct parental involvement declines and gradually transitions to monitoring and supervision of those activities (Berg et al., 2017). Even though diminishing parental involvement is in accordance with normative development, decreased parental involvement has been associated with reduced diabetes self-care and glucometrics over time, which is why the importance of ongoing parental engagement has been stressed (Ellis et al., 2007a; Ingerski et al., 2010; King et al., 2012, 2014).

For parents, each developmental period presents unique challenges in their diabetes care involvement. Young children, for example, show unpredictable physical activity patterns and changing food preferences, which complicates management of mealtime behaviors (Tully et al., 2018) and hypoglycemia prevention (Markowitz et al., 2015). School-aged children with T1D require parents to navigate their child’s increased independence and broadened social environment, which involves reinforcing their child’s diabetes self-care activities and transferring responsibilities to other adults (Markowitz et al., 2015). During adolescence, challenges in parent–child collaboration intensify as physiological changes and the growing influence of peers often impede diabetes care (Chiang et al., 2018; Markowitz et al., 2015). Whereas sustained parental involvement remains crucial during this phase, it often conflicts with their child’s increasing need for autonomy and peer identification (Chiang et al., 2018; Jaser, 2011), making families prone to conflicts.

Across all these developmental phases, a collaborative parenting style is crucial, where the parents attune their support to their child’s (changing) needs (Wysocki et al., 2009). Collaborative parenting has been related to higher quality of life and lower HbA1c (Gruhn et al., 2016; Weissberg-Benchell et al., 2009). However, many families have difficulty navigating this balance. Some parents use well-intentioned yet fear-driven parenting strategies, including criticism, blaming, and nagging (Ivey et al., 2009; Weinger et al., 2001). These intrusive parenting behaviors are related to decreased child well-being (Gruhn et al., 2016; Tilden et al., 2024; Weissberg-Benchell et al., 2009) and suboptimal glucometrics (Cameron et al., 2008; Lewin et al., 2006). Other parents disengage from diabetes care prematurely to minimize conflicts and hassles, or as a result of stress (Palmer et al., 2004), yet these permissive or uninvolved parenting strategies are also related to suboptimal outcomes (Shorer et al., 2011; Wiebe et al., 2005).

To address parental behavior toward the child, parenting interventions have been developed, here defined as any intervention delivered to parents that aims to modify their behavior toward their child. Parenting interventions are presumed to impact child’s health outcomes both directly—through targeting ineffective parenting strategies—and indirectly—by reducing family-related stress (Morawska et al., 2015). While there is a substantial overlap in content between parenting programs in T1D, they vary greatly in aims, intensity, and delivery. Some programs target vulnerable families through intensive interventions (Ellis et al., 2005a; Wysocki et al., 2006) or brief programs (Ellis et al., 2017), while other programs address family dynamics in a broad population through preventive parenting programs (Anderson et al., 1999; Laffel et al., 2003) or programs with a broader scope (e.g., education) (Kichler et al., 2013; Murphy et al., 2012).

Previous reviews have examined these interventions, yet a recent and comprehensive overview of T1D parenting interventions is currently lacking for several reasons. First, existing reviews collated findings of parenting interventions in T1D with other types of interventions (Hilliard et al., 2016; Hood et al., 2010; Winkley et al., 2020) or other pediatric conditions (Mitchell et al., 2020; Morawska et al., 2015). However, tailoring interventions to diabetes has been considered essential and distilling their key (effective) ingredients is necessary to impact clinical practice (Hood & Nansel, 2007). Second, these reviews were either not systematically conducted (Hilliard et al., 2016; Hood & Nansel, 2007; Morawska et al., 2015) or limited their scope to specific populations [young children (Lohan et al., 2015)], study designs [randomized-controlled designs (Feldman et al., 2018; Ispriantari et al., 2023; Law et al., 2019; Winkley et al., 2020; Zhao et al., 2019)], or outcomes [HbA1c (Winkley et al., 2020); parental psychosocial adjustment (Zhao et al., 2019)]. These focused approaches risk overlooking key studies that could provide valuable insights. Third, rapid technological advancements introduce new parenting challenges (Brew-Sam et al., 2021), which may have prompted the development of new parenting interventions since the publication of previous reviews. Finally, reviews of parenting in T1D have generally examined parenting interventions broadly without addressing specific behavioral techniques. As behavioral techniques have been related to effectiveness in parenting interventions in the field of attention-deficit hyperactivity disorder (Hornstra et al., 2023), examination of their contribution to effectiveness in the context of T1D is warranted.

Altogether, this calls for a systematic review that provides a comprehensive assessment of parenting interventions in T1D and delineation of their respective components. This systematic review (1) summarizes and appraises the effectiveness of parenting interventions in pediatric diabetes on outcomes related to family dynamics (e.g., conflict), parental well-being (e.g., distress), child well-being (e.g., quality of life), and diabetes (e.g., HbA1c) and (2) categorizes interventions by their active components. As family dynamics constitute an important modifiable risk factor in diabetes care—alongside many non-modifiable risk factors (e.g., socio-economic status)—these findings are valuable to inform the implementation of effective interventions to improve psychosocial and diabetes outcomes in clinical practice.

## Methods

This review was preregistered in PROSPERO (CRD42022356654) and follows PRISMA standards (Page et al., 2021); the PRISMA checklist is available as Supplementary File S1.

### Search strategy

On September 9, 2022 [updated on January 17, 2024 and February 25, 2025 (Bramer & Bain, 2017)], all five databases were searched for articles published since 1980: PubMed, EMBASE, Cochrane Library, PsycInfo, and CINAHL. Search terms included four categories of words related to diabetes, children/adolescents, parents/caregivers, and interventions. We did not include outcomes as search terms to avoid missing relevant studies, as the phrasing of psychosocial outcomes varies across papers, and this could limit search results. All search terms were adapted to each database to meet their requirements (full search strategy available in Supplementary File S2). Cross-referencing was completed for the included full-text reports. Reviews related to the current systematic review were also searched for relevant original studies (Feldman et al., 2018; Hilliard et al., 2016; Hood & Nansel, 2007; Hood et al., 2010; Ispriantari et al., 2023; Law et al., 2019; Lohan et al., 2015; Mitchell et al., 2020; Morawska et al., 2015; Winkley et al., 2020; Zhao et al., 2019). During the reference list searching of included articles and related reviews, some initially excluded reports were re-assessed for eligibility based on full text. Additional information was requested from the authors if eligibility was unclear.

### Eligibility criteria

Eligibility criteria for studies were: (1) intervention study; (2) population of children/adolescents (0–18 years) diagnosed with T1D for ≥6 months (as formal eligibility criterion or average of sample); (3) outcomes reported as quantitative change (≥2 time points, including at least pre- and post-intervention) in family dynamics/parenting behavior (e.g., self-reported or observed communication, conflict or parenting behavior/involvement), parental well-being (e.g., parent mental health, beliefs, adjustment), child well-being (e.g., quality of life, diabetes distress), and/or diabetes outcomes (self-reported or objective measures of diabetes, such as HbA1c, self-management, blood glucose monitoring frequency); and (4) peer-reviewed full texts or conference abstracts/dissertations written in English. To ensure comprehensiveness of the review, we included findings of all studies regardless of study designs (e.g., uncontrolled and pilot/feasibility studies were also included). However, as pilot/feasibility study designs might be underpowered to detect potential differences, we prioritized results of full-scale trials over pilot/feasibility designs when describing study findings. Interventions were eligible if they: (1) addressed parenting behavior/family dynamics (as the sole focus or embedded in broader programs); (2) were healthcare-delivered or self-directed; and (3) were ≥50% delivered to one or more parent(s)/caregiver(s) residing with the child. Studies were excluded if intervention content solely focused on improving medical procedures or reducing parental distress, without targeting parenting behavior.

### Study selection

After EndNote exportation and deduplication (Bramer et al., 2016), M.J. and G.N. independently screened all records for eligibility based on title and abstract. We used ASReview—an open-source AI-aided tool using active learning techniques—to facilitate and accelerate the screening process (van de Schoot et al., 2021). ASReview shuffles the order of record presentation based on previous eligibility decisions made by the reviewer, ensuring that the most relevant papers are presented next. This allows identification of relevant references at an early stage of the screening process, while the reviewer remains in charge of all eligibility decisions. Each reviewer entered identical prior knowledge to train the model and consequently screened all records; no stopping criteria were applied. Results were compared, and disagreements were resolved through discussion. Full-texts articles were assessed by M.J. (together with G.N. if inconclusive).

### Data extraction and synthesis

M.J. completed data extraction. G.N. additionally performed independent duplicate data extraction in 10% of included reports. These 10% were purposively selected by M.J. based on the high complexity of data extraction. Double data extraction for all papers was not feasible due to resource constraints. However, G.N. was consulted throughout data extraction (in 73% of reports), which helped establish data extraction rules to ensure consistent data extraction across the papers (Table 1). Discrepancies were resolved through discussion. Extraction included participant characteristics (*N*, sex, race, baseline HbA1c), inclusion criteria, and intervention characteristics (e.g., name, intensity, delivery mode, estimated % targeting parenting; Table 2). To estimate the percentage of the intervention that was delivered to parents and targeted parenting behavior, M.J. and G.N. jointly examined the program material during structured meetings. Estimates were based on the number of topics addressing parenting behavior compared to the total number of reported topics, or—if the manual was provided—based on the number of pages compared to the total number of pages (Table 2). Findings of post- and first follow-up outcomes (*p*-values, effect sizes, online Supplementary Table S1) were extracted and grouped into four outcome categories: family dynamics/parenting behavior, parental well-being, child well-being, and diabetes. If data were not described, the description was set as “not reported” (Table 2, Supplementary Tables S1 and S2). For significant findings on objectively measured diabetes outcomes, means were extracted and described in the text if available (e.g., HbA1c, blood glucose monitoring frequency). We grouped studies according to intervention similarities and presented results per study (collating multiple reports) (Li et al., 2024). If reported, intervention effects for specific subgroups (either through subgroup or moderation analyses) were extracted (Supplementary Table S2). No sensitivity analyses were conducted, nor did we assess overall certainty in the evidence due to heterogeneity in extracted outcomes.

**Table 1.** Decision rules for data extraction.

Scenario	Extracted data
Multiple analyses were conducted	Results from pre-specified or more advanced analyses (interaction effects in RM-ANOVA/multilevel analyses) were prioritized over pre–post *t*-tests. Results of unadjusted analyses were prioritized over adjusted analyses. Post-hoc contrasts were only considered if the overall effect of interest (e.g., interaction effect) was significant.
Outcomes were operationalized in multiple ways	Outcomes were extracted that were pre-specified or, if unavailable, continuous (as opposed to dichotomized/adapted/combined outcomes) If findings for both total and subscale scores were presented, only findings of total scores were extracted.
Multiple measures were used to assess the same construct	The measure that has the best psychometric properties is best recognized or is most frequently used in the field was extracted
The paper did not report effects that were relevant for their design/this review’s research questions (e.g., only reporting pre–post changes within intervention condition despite having a control condition; only presenting results for attendees instead of overall group effects)	Effects were described as “not reported” (NR)
The results section omitted findings of an outcome that was described in the methods section	Effects were described as “not reported” (NR)
Findings of a study were described in both a conference abstract/dissertation and full-text published paper.	Findings of the published paper were included; the conference abstract/dissertation was excluded.

**Table 2.** Intervention and study characteristics of included reports.

	First author, year	Name intervention	Intensity	Sample characteristics (*N*, child’s sex and race, HbA1c)	Relevant inclusion criteria (demographic characteristics, diabetes duration, clinical subgroup)	Mode of delivery: family/parent(s)–child/parent(s); individual/group; joint/parallel (for parent–child)	Estimated % of intervention targeted at parenting***
Family systems therapy	Wysocki, 1999, Wysocki, 2000, Wysocki, 2001	Behavioral family systems therapy (BFST)	10 sessions,* duration NR in 3 months	*N* = 119 (I = 38, AC = 40, CAU = 41) Male = 42%, Caucasian = 78% HbA1c = 11.8(3.1)	12–16.75 years, diabetes duration ≥12 months, elevated parent–child conflict	Family, individual	100%
	Harris, 2003, Harris, 2005	Behavioral family systems therapy (BFST)	10 sessions* session duration NR in 5–8 weeks	*N* = 18 Male = 67%, Caucasian = 67% HbA1c = 11.4 (1.4)	13–18 years, two consecutive HbA1c ≥9.0 or ≥2 missed visits and most recent HbA1c ≥9	Family, individual	100%
	Wysocki, 2006, Wysocki, 2007, Wysocki, 2008	Behavioral family systems therapy—diabetes (BFST-D)	12 sessions,* session duration NR in 6 months	*N* = 104 (I = 36, AC = 36, CAU = 32) Male = 55%, Caucasian = 63% HbA1c = 9.6 (1.6)	11–16 years T1D or insulin-treated T2D, diabetes duration ≥24 months, HbA1c ≥8.0	Family, individual	100%
	Harris, 2015 Riley, 2015, Duke, 2016	Behavioral family systems therapy—diabetes (BFST-D) (Skype and face-to-face)	10 sessions* 60–90 min in 12 weeks	*N* = 90 Male = 61%, Caucasian = 88% HbA1c = 11.1(1.7)	12–19 years, diabetes duration ≥12 months, HbA1c ≥9.0	Family, individual	100%
	Lehmkuhl, 2010	Telehealth behavioral therapy (TBT) (phone)	36 sessions,* 15–20 min in 12 weeks	*N* = 32 (I = 18, WC = 14) Male = 28%, Caucasian = 81% HbA1c = 10.6(2.0)	9–17 years, diabetes duration ≥6 months, HbA1c > 9%	Parent(s)–child, individual, joint	100%
	Salcudean, 2024	Systemic family psychotherapy	12 sessions,* 90 min in 6 months	*N* = 64 (I = 22, AC = 22, CAU = 20), Male = 64%, race = NR HbA1c = 8.4 (1.5)	Diabetes duration ≥10 months HbA1c >7 %	Family, individual	∼60%
Multisystemic therapy	Ellis, 2004	Multisystemic treatment (MST)	Varying, mean = 46 sessions* variable session duration in 6.5 months	*N* = 25 (I = 13, CAU = 12) Male = 56% African American = 61% HbA1c = 17.6(3.3)	Diabetes duration ≥ 12 months GHb ≥ 13%	Family, individual	100%
	Ellis, 2005a Ellis, 2005b Ellis, 2007b Ellis, 2007c Naar-King, 2007	Multisystemic treatment (MST)	Varying, mean = 48 (19) sessions for completers and 9 (8) for non-completers* variable session duration in 6 months	N = 127 (I = 64, CAU = 63) Male = 49% African American = 63% HbA1c = 11.3(2.3)	10–17 years diabetes duration ≥ 12 months HbA1c ≥ 8% prior year (average) and most recent	Family, individual	100%
	Ellis, 2012	Multisystemic treatment (MST)	Varying, mean = 46 (19) sessions* variable session duration in 6 (1) months	*N* = 146 (I = 74, AC = 72) Male = 44%, African American = 77% HbA1c = 11.7(2.5) T1DM = 90%, T2DM = 10%	10–18 years T1DM or T2DM diabetes duration ≥12 months HbA1c ≥8% prior year (average) and most recent)	Family, individual	100%
	Ellis, 2019	REACH for Control (RFC)	Varying, estimated is 36 sessions* 30–90 min in ∼6 months	*N* = 50 (I = 26, CAU = 24) Male = 38%, African-American = 79% HbA1c = 11.5 (1.9)	10–18 years diabetes duration ≥12 months HbA1c ≥9% prior year (average) and most recent)	Family, individual	100%
Routine care integrated	Anderson, 1999	Office teamwork intervention (Office TW)	4 sessions* 20–30 min in 1 year	*N* = 82 (I = 28, AC = 30, CAU = 24) Male = 50%, race = NR HbA1c = 8.5 (1.1)	10–15 years diabetes duration >12 months HbA1c between 6.6 and 10.4	Parent(s)–child, individual, joint	∼55%
	Laffel, 2003	Teamwork intervention (TW)	4 modules* 15–20 min in 1 year	*N* = 100 (I = 50, CAU = 50) Male = 53%, race = NR HbA1c = 8.4 (1.7)	8–17 years diabetes duration >2 months and ≤6 years	Parent(s)–child, individual, joint	∼40%
	Svoren, 2003	Family psychoeducation + care ambassador (Family PE + CA)	8 sessions* 15–30 min in 2 years	*N* = 299 (I = 97, AC = 94, CAU = 108) Male = 43%, race = NR HbA1c = 8.7 (1.2)	7–16 years diabetes duration ≥6 months	Parent(s)–child, individual, joint	∼20%
	Katz, 2014	Family psychoeducation + care ambassador (Family PE + CA)	∼8 sessions* 30 min in 2 years	*N* = 153 (I = 50, AC = 52, CAU = 51) Male = 44%, White = 91% HbA1c = 8.4 (1.4)	8–16 years diabetes duration ≥6 months	Parent(s)–child, individual, joint	∼25%
	Holmes, 2014	Family teamwork coping skills training (Family TW CST)	4 sessions* 30–45 min in 1 year	*N* = 226 dyads (I = 137, AC = 89) Male = 48%, White = 71% HbA1c = NR	11–14 years diabetes duration >12 months	Parent(s)–child, individual, joint	∼45%
	Murphy, 2007	FACTS	4 sessions* 60 min in 1 year	*N* = 78 (I = 33, WC = 34)* Male = 56%, race = NR HbA1c = 9.1 (1.3)	6–16 years diabetes duration ≥12 months	Parent(s)–child, group, joint	∼50%
	Murphy, 2012	FACTS	6 sessions* 90 min in 6 months	*N* = 305 (I = 158, CAU = 147) Female = 52%, White = 93% HbA1c = 9.3 (1.9)	Diabetes duration ≥12 months	Parent(s)–child, group, joint	∼55%
	Nansel, 2009	WE-CAN	∼3 sessions* session duration NR in 1 year (average 2.7 visits)	*N* = 122 (I = 60, CAU = 62) Male = NR, White 71.1% HbA1c = 8.4(SD NR)	9–14.5 years diabetes duration ≥12 months HbA1c <13.0%	Parent(s)–child, individual, joint	100%
	Nansel, 2012, Nansel, 2015, Gee, 2017, Temmen, 2022, Lu, 2023	WE-CAN	∼6–8 sessions* ∼30 min in 2 years	*N* = 390 (I = 201, CAU = 189) Male = 51%, White = 75% HbA1c = 8.4 (1.2)	9–15 years diabetes duration ≥3 months HbA1c 6.0–12.0 (or >6.0 if diagnosed <12 months)	Parent(s)–child, individual, joint	100%
	Monaghan, 2015	Checking in	1 session* mean length 3 min	*N* = 30 Male = 53%, Caucasian = 67% HbA1c = 8.9 (1.6)	11–15 years diabetes duration ≥12 months	Parent(s)–child, individual, joint	∼70%
	Ellis, 2017	Computer-delivered motivational intervention (3Ms)	3 sessions* session duration NR in 6 months	*N* = 67 (I = 19, AC = 24)* Male = 44%, African American = 100% HbA1c = 10.6 (2.2)	10–14 years diabetes duration ≥6 months African American (self-identified)	Parent, individual	100%
	Ellis, 2024, Knauft, 2024	Computer-delivered motivational intervention (3Ms)	1–3 sessions (depending on# clinic visits)* 10–20 min in 1 year	*N* = 149 (I = 75, CAU = 74) Male = 42%, Black = 100% HbA1c = 11.5 (2.7)	10–14 years diabetes duration ≥6 months Black (self-identified)	Parent, individual	100%
Combined parent-child	Ambrosino 2008, Grey, 2009, Grey, 2011**	Coping skills training (CST)	6 sessions* 90 min in 6 weeks	*N* = 111 (I = 65, AC = 46) (Grey 2011: *N* = 181 (I = 75, C = 106) Male =39%, White = 85% HbA1c = 7.0 (1.3)	8–12 years/≤12 years (Grey 2011) diabetes duration ≥6 months	Parent(s)–child, group, parallel	∼30%
	Opipari, 2005 *(conference abstract)*	Kicking in diabetes support (K.I.D.S.)	6 sessions* duration NR in 6 weeks	*N* = 29 Male = NR, race = NR HbA1c = NR	10–14 years HbA1c >7.5 Receiving Medicaid	Parent(s)–child, group, parallel and joint	NA
	Kichler, 2013	Kicking in diabetes support (KIDS)	6 sessions* 50–75 min in 6 weeks	*N* = 30 (I = 14, WC = 16) Male = 47%, Caucasian = 77% HbA1c = 10.0 (2.1)	13–17 years diabetes duration ≥6 months	Parent(s)–child, group, parallel and joint	∼70%
	Kichler, 2021	Kicking in diabetes support (KIDS)	8 sessions* 60 min (incl. 2 booster sessions) in period NR	*N* = 38 Male = 25%, Caucasian = 80% HbA1c = 9.3 (1.7)	10–17 years diabetes duration ≥6 months	Parent(s)–child, group, parallel and joint	∼70%
	Satin, 1989	Multifamily group intervention (MF), with parental diabetes simulation (MF+S)	6 sessions* 90 min in 6 weeks	*N* = 32 families (MF = 11, MF+S = 12, CAU = 9) Male = 38%, race = NR HbA1c= 13.0 (1.2)	12–19 years	Parent(s)–child, group, joint	∼65% for both groups
	Carpenter, 2014	Multifamily group problem solving intervention (MF group PS)	4 sessions* 75 min in 4 weeks	*N* = 67 Male = 42%, Caucasian = 69% HbA1c = 10.1 (1.9) T1DM = 92.5%; T2DM = 7.5%	T1DM or insulin treated T2DM	Parent(s)–child, group, joint	100%
	Patel, 2019 *(conference abstract)*	Counseling	6 sessions* duration NR in 6 months	*N* = 37 Male = 54%, race = NR HbA1c = NR	12–18 years	Parent(s)–child, individual, joint	NA
Stand-alone parent training	Doherty, 2013	Triple P (Level 4, self-directed)	Own pace ∼ 10* 60 min in 10 weeks	*N* = 79 (I = 42, CAU = 37) Male = 64%, race = NR HbA1c = 8.6 (1.3)	11–17 years no mental health diagnoses	Parent(s), individual	100%
	Westrupp, 2015	Triple P (level 4)	10 sessions* 60 min in 10 weeks	*N* = 76 (I = 38, CAU = 38) Male = 57%, race = NR HbA1c = 8.0(0.9)	4–12 years diabetes duration ≥6 months	Parent(s), individual	100%
	Arkan, 2020	Triple P (level 4)	8 sessions* 30–120 min in 8 weeks	*N* = 32 Male = NR, race = NR HbA1c = 8.2(SD NR)	3–12 years Non-divorced	Parent(s), group	100%
	May, 2017	Individualized feedback intervention	1 session* mean length 19 (4) min	*N* = 79 (I = 39, AC = 40) Male = 44%, White = 89.9% HbA1c = NR	13–18 years	Parent(s), individual	100%
	Saßmann, 2012	DELFIN parenting program	5 sessions* 120 min in 6 weeks	*N* = 37 (I = 19, WC = 18) Male = NR, race = NR HbA1c = 7.2(0.6)	2–10 years	Parent(s), group	100%
	Mitchell, 2024	Healthy living Triple P	2 sessions* 120 min in 2 weeks	*N* = 50 (I = 22, CAU = 28) Male = 28%, race = NR HbA1c in range (<7.5%) = 44%	2–10 years diabetes duration ≥3 months, concerns about child	Parent(s), group	100%
	Jones, 2024	Psychoeducation for parents to prevent disordered eating (PRIORITY)	2 sessions* 120 min in 2 weeks	*N* = 89 (I = 44, WC = 45) Male = 61%, White = 90% HbA1c = 7.5 (3.9)	11–14 years	Parent(s), group	100%
	SENCE Study Group, 2021; Commissariat, 2023, Van Name, 2023	Continuous glucose monitoring (CGM) + Family Behavioral Intervention (FBI)	5 sessions* 30 min in 19 weeks	*N* = 143 (I = 50, C = 44)* Male = 51%, White = 69% HbA1c = 8.2(0.8)	2–8 years diabetes duration ≥3 months HbA1c 7.0–10.0, not using CGM	Parent(s), individual	∼20%
	Rothman-Kabir, 2022	New authority training	10 sessions* session duration NR in 10 weeks	*N* = 36 Male = 72.2%, race = NR HbA1c = NR	12–18 years diabetes duration ≥12 months, HbA1c > 8% past 6 months	Parent(s), individual	100%
	Jaser, 2018	Communication and coping intervention	7 sessions* session duration NR in 3 months	*N* = 30 (I = 15, CAU = 15) Male = 62%, White = 100% HbA1c = 9.0 (1.0)	10–16 years diabetes duration ≥12 months increased maternal distress/depressive symptoms	Parent(s), individual	∼30%
	Kawamura, 2012 *(conference abstract)*	Motivational interviewing for parents	6 sessions*duration NR in period NR	*N* = 33 (I=NR, C=NR) Male = NR, race = NR HbA1c = NR	HbA1c > 7.5	Parent(s), group	NA
	Liberman, 2017 *(conference abstract)*	Parental authoritativeness intervention	5 sessions* duration NR* period NR	*N* = 33 (I = 9, AC = 9, CAU = 15) Male = NR, race = NR HbA1c = NR	10–18 years HbA1c > 8	Parent(s), group	NA
Contracting	Hannon, 2018, Halper, 2022	Family goal setting	2 session* 30 min in 3 months	*N* = 33 Male = 49%, White = 85% HbA1c = 9.0 (1.9)	12–18 years diabetes duration ≥6 months	Parent(s)–child, individual, joint	∼50%
	Carroll, 2011	Behavioral contracting *(coincides with other intervention—“cell phone glucose meter”*)	2 sessions* 20 min in 3 months + text reminders	*N* = 10 Male = 50%, race = NR HbA1c = NR	14–18 years	Parent(s)–child, individual, joint	∼50%
	Stanger, 2013	Contingency training	14 sessions* 60 min in 14 weeks	*N* = 17 Male = 29%, Caucasian = 71% HbA1c = 11.6 (2.5)	12–17 years diabetes duration ≥18 months HbA1c ≥ 8% prior 6 months and most recent	Parent(s)–child, individual, parallel	∼50%
	Epstein, 1981	Behavioral treatment	8 sessions* 60–90 min in 12 weeks	*N* = 19 Male = NR, White = NR HbA1c = 10.0 (SD NR)	8–12 year No hospitalizations during prior year	Parent(s)–child, individual, parallel	∼10%
Digital	Hilliard, 2020	Type 1 Doing Well (app)	At own pace, access to app for 3–4 months	*N* = 80 (I = 55, CAU = 25) Male = 41%, White = 61% HbA1c = 9.0 (2.1)	12–17 years diabetes duration ≥6 months	Parent(s), individual	100%
	Whittemore, 2020	Type 1 Teamwork eHealth program	At own pace, 6 interactive sessions* session duration NR in 3 months	*N* = 162 (I = 81, WC = 81) Male = 47%, White = 91% HbA1c = 7.9 (1.2)	11–16 years	Parent(s), individual	∼65%
Young children	Tully, 2018	Type One Training (TOT) *(coincides with start CGM for 6/10 participants)*	6 sessions* duration NR in 12 weeks	*N* = 10 Male = 60%. White = 50% HbA1c = 8.1 (0.9)	2–5 years diabetes duration ≥12 months parent ≥21 years	Parent(s), individual and group	∼30%
	Mackey, 2022	Type One Training (TOT)	6 sessions* 40–90 min in 9–12 weeks	*N* = 36 (I = 19, CAU = 17) Male = 63.9%, White = 74% HbA1c = 8.2 (1.0)	2–5 years diabetes duration ≥12 months parent ≥ 21 years	Parent(s), individual and group	∼30%
	Patton, 2014	BEST MEALS	6 sessions* session duration NR in 6 weeks	*N* = 9 Male = 33%, White = 89% HbA1c = 8.2 (1.3)	2–6 years diabetes duration ≥6 months	Parent(s), group	∼45%
	Patton, 2020	REDCHiP	10 sessions* 30–60 min in 10 weeks	*N* = 43 (I = 22, WC = 21) Male = 60%, Caucasian = 95% HbA1c = 8.1(1.0)	1–6 years diabetes duration ≥6 months	Parent(s), individual and group	∼15%

First author and year of publication are displayed. Sample sizes are based on number of participants initially randomized to each group (rather than numbers analyzed), unless the former is not available. Numbers between brackets represent *SD*.

* Numbers of subgroups do not add up to total, because the total sample size includes a group that is irrelevant for our research question (effects were not extracted).

** Grey (2011) combined data of two different trials.

*** For interventions described in full-text papers, M.J. and G.N. jointly estimated the percentage of the intervention that is delivered to parents and targeted parenting behavior/family dynamics based on the number of topics compared to the total, or—if the manual is available—the number of pages targeting parenting as compared to the total number of pages. Percentages were rounded per 5%.

NR = not reported; NA = not available; I = intervention; AC = active control, includes all control conditions where parents receive an alternative intervention; CAU = care-as-usual; WC = waitlist control; CGM = continuous glucose monitoring; incl. = including; GHb = total glycohemoglobin.

In order to shed light on the programs’ active components, we coded active intervention components with a hybrid taxonomy, explained below. Parenting interventions involve two behavioral change processes: (1) behavioral techniques used to motivate parents to change their own behavior, and (2) behavioral strategies taught to parents to change their child’s behavior. The Behavioral Change Technique (BCT) Taxonomy (Michie et al., 2013) may be suitable to code the first process, but is less suitable to structure the second process. For the latter, other taxonomies structure parenting strategies in programs for disruptive child behaviors (Hornstra et al., 2023; Leijten et al., 2019), but they do not capture unique features of programs for chronic conditions like T1D (e.g., teaching parents distraction strategies during uncomfortable medical procedures or parental simulation of diabetes to enhance empathy). For the current review, we therefore adapted the aforementioned taxonomies and tailored them to our field using inductive coding.

As a result, the taxonomy differentiates between *what* parents are taught to manage child behavior [primarily based on taxonomies from Hornstra et al. (2023) and Leijten et al. (2019), relating codes to the BCT Taxonomy where possible] and *how* parents are motivated to change parenting behavior (primarily based on BCT Taxonomy). The codebook is available at Open Science Framework (OSF) (Jansen et al., 2024). An example code in the *What* category includes *Social reward* (“Parents are trained in the administration of social rewards to promote desired behaviors. This can involve praise, encouragement, affection, or physical proximity.”); an example code in the *How* category includes *Behavioral practice* [“Opportunities (in-session/outside of session) for parents to practice skills through rehearsal or role-playing situations with their partner, other parents, their child and/or professionals”]. Coding was based on requested supportive documentation (program manuals) or—if unavailable—on information from the included report(s) and/or papers describing intervention development.

### Risk of bias assessment

For full-text reports, we assessed risk of bias with the Cochrane Risk of Bias (RoB) tool [randomized controlled trials (RCTs); Sterne et al., 2019] and ROBINS-I (non-randomized studies; Sterne et al., 2016). For both tools, review-specific implementation documents (Minozzi et al., 2022) were created jointly between M.J. and G.N. to enhance consistency across assessments (Jansen et al., 2024). Decision rules in these documents were based on issues encountered in the included papers—discussed between M.J. and G.N.—and calibrated with another research group. The implementation documents were created and continuously updated based on new complexities identified, with prior assessments being revised accordingly. Using these documents, M.J. performed all quality assessments, G.N. advised as needed (in 44% of assessed reports), and duplicately assessed independently 10% of reports that were selected based on high ambiguity. Discrepancies were resolved through discussion. The robvis visualization tool was used to create traffic plots (McGuinness & Higgins, 2020).

### Study protocol deviations

The tables include parsimonious study characteristics. High study volume and restricted team resources prevented forward citation tracking and GRADE assessment, and necessitated one-reviewer (M.J.) full-text screening and RoB assessment. However, we ensured consistency by frequent consultation with Reviewer 2 (G.N.), the establishment of decision rules in data extraction and risk of bias assessments (Jansen et al., 2024), and 10% duplicate RoB assessment. Outcome heterogeneity precluded statistical data synthesis. To elucidate intervention–report interrelations, study findings were ordered accordingly, irrespective of RoB assessment.

## Results

### Reports/studies

The search yielded 72 eligible reports (68 reports, 4 conference abstracts) about 51 individual studies (*k* = 51) (Supplementary Figure S1). Findings of a single trial were often dispersed across multiple reports. Thirty-seven studies used a RCT design (*k* = 13 pilot/feasibility studies), and 14 used an uncontrolled design (*k* = 8 pilot/feasibility studies) (Table 2). Some studies (*k* = 17) specifically targeted a clinical subgroup with elevated baseline HbA1c, familial conflict, parental distress, or child concerns. Most studies (*k* = 28) targeted (pre)adolescents (≥10 years), a few (*k* = 4) focused on very young children (≤6 years), while the rest included an intermediate, mixed, or undefined age span. A total of 37 interventions were evaluated, often with overlapping content. Treatment intensity ranged from 1 to 46 sessions; estimates of intervention content targeting parenting behavior ranged from 10% to 100%. Most interventions were delivered to parents and children/the entire family (*k* = 31); others solely targeted parents (*k* = 20). Interventions were delivered in group format (*k* = 15), individually (*k* = 33), or a combination thereof (*k* = 3). Supplementary Tables S1 and S2 report study findings and additional analyses, respectively.

### Family systems therapy (3 interventions, 6 studies, 13 reports)

Behavioral family systems therapy (BFST) consists of four components (problem-solving training, communication skills training, cognitive restructuring, family therapy; 10–12 individual sessions), whereas systemic family psychotherapy incorporates an eclectic mix of 12 approaches (12 sessions). Both are tailored to families of youth with suboptimal diabetes management or increased levels of diabetes-related conflict.

In a first trial, BFST (10 sessions over 3 months) (Wysocki et al., 1999, 2000, 2001) sustainably decreased diabetes-specific conflicts, some measures of parent–child relationship, and some maternal communication behaviors compared to both educational control and care-as-usual. General conflicts did not change or only short-term effects (immediately post-intervention). Effects for other family and child outcomes were absent or less robust. Diabetes outcomes did not improve, although some age- and gender-specific effects were found for HbA1c.

Next, a small-scale, uncontrolled pilot study assessed the effects of in-home BFST (Harris et al., 2005; Harris & Mertlich, 2003). Whereas mothers reported short-term decreases in conflicts, the effects were not maintained and not confirmed by other respondents. Again, no effects were found for child adjustment or diabetes outcomes, although the sample size may have been too small to detect changes in outcomes.

A second trial compared a diabetes-specific version of BFST (BFST-D) to an educational control condition and care-as-usual (Wysocki et al., 2006, 2007, 2008). Adaptations included diabetes-specific elements (e.g., parental diabetes simulation, behavioral contracting) and two additional sessions to impact diabetes outcomes. Nevertheless, overall post-intervention effects of BFST-D on HbA1c (pre: 9.6%; post: 8.8%) were absent compared with an educational control (pre: 9.7%, post: 8.9%) and inconsistent between reports compared with the care-as-usual condition [pre: 9.5%, post: 9.2%, not significant according to (Wysocki et al., 2006); pre: 9.6%, post: 9.1%, significant according to Wysocki et al. (2007)]. Among those with elevated baseline HbA1c (>9.0%), HbA1c improved (−1.3%) when compared with care-as-usual (−0.4%), but not with the educational control (−1.1%). Regarding self-reported diabetes self-care (i.e., following treatment recommendations, formerly known as “adherence”), overall effects were also not significant, although interactions showed some improvements within both conditions of low (≤9.0%) and high (>9.0%) baseline HbA1c levels. Among those with elevated baseline HbA1c, families in the BFST-D condition showed a decrease in conflicts compared to an increase in both control conditions. Regarding family dynamics, similar patterns emerged as in the initial trial: sustained improvements in communication were most evident for mothers (as opposed to fathers and children) and some, but not all, family interaction behaviors. Parent–child relation outcomes did not improve.

In a third study, BFST-D delivered through Skype was compared to face-to-face delivery (Duke et al., 2016; Harris et al., 2015; Riley et al., 2015), with similar effectiveness on both self-reported outcomes and HbA1c (pre, post, and follow-up for face-to-face: 11.1%, 10.5%, 10.3%; for Skype: 11.2%, 10.4%, 10.6%).

The BFST(-D)-inspired Telehealth Behavior Training (TBT) was delivered through phone in more frequent sessions with shorter durations in a pilot trial (Lehmkuhl et al., 2010). Compared to a waitlist control condition, TBT participants did not report improvements in family dynamics or diabetes outcomes. On the contrary, TBT participants reported decreases in warmth and increases in unsupportive parenting, suggested to result from increased (quantitative) parental involvement in diabetes management. However, the small sample size of the pilot design precluded detection of significant changes.

Finally, a recent systemic family psychotherapy intervention was compared with individual therapy and care-as-usual (Salcudean & Lica, 2024). HbA1c decreased in the family therapy (pre: 8.6%, post: 7.5%) compared to an increase in the care-as-usual condition (pre: 8.2%, post: 8.9%) and no effect in the individual therapy condition (pre: 8.4%, post: 8.0%). Conflicts and closeness improved when compared with care-as-usual, while contrasts with the individual therapy condition were unclear.

Overall, BFST(-D)—either delivered face-to-face or through Skype—appears partially successful in reducing (diabetes-) conflicts and improving maternal and familial communication behavior, with many effects persisting over time after diabetes-specific components were added to the intervention. Findings regarding paternal or child behavior yielded mixed results, whereas effects on diabetes outcomes were only found in specific subgroups and comparison groups. A phone-delivered adapted BFST intervention was not powered to detect changes in outcomes, while another systemic family psychotherapy impacted HbA1c and family outcomes.

### Multisystemic therapy and related interventions (2 interventions, 4 studies, 8 reports)

Multisystemic therapy (MST)—initially developed for adolescents with severe antisocial behavior—utilizes multiple intervention techniques (including cognitive-behavioral therapy, parent training, and BFST). It follows overall treatment principles rather than a session-by-session manual, is family-tailored, and addresses multiple systemic contexts of the child beyond the individual and parent–child relationship. MST is the most intensive, outreaching, and individualized intervention for families with substantial diabetes management difficulties (an average of 48 sessions over 6 months).

In a large RCT, MST reduced the 6-month window of hospital admissions (pre: 0.4, post: 0.1) and DKA admissions (pre: 0.5, post: 0.2) compared to care-as-usual (pre and post for hospital admissions: 0.4, 0.5; DKA admissions: 0.4, 0.6) (Ellis et al., 2005a, 2005b, 2007b, 2007c; Naar-King et al., 2007). Increased daily blood glucose monitoring frequency was reflected in both self-reported and objectively measured outcomes (pre and post for MST: 1.8, 2.5; care-as-usual: 2.2, 2.0), although these effects were only maintained in two-parent families. Effects on HbA1c were described in several reports of this trial, with inconsistent findings. While two reports did not report an effect on HbA1c (only trends) (Ellis et al., 2005a, 2007c), one report described a stable HbA1c in the control condition for the three time points (11.3%, 11.3%, 11.1%), but a decreasing HbA1c for MST (pre: 11.4%, post: 10.7%) that was not maintained at follow-up (11.0%) (Ellis et al., 2007b). Regarding family outcomes, effects on caregiver support post-intervention were found for both caregivers in two-parent but not single-parent families. General measures of family relationships did not change, while parental overestimation of their child and child diabetes distress decreased. Family measures and diabetes distress among adolescents did not mediate treatment effects of MST on glucose monitoring or HbA1c; rather, MST appeared to have a direct effect on glucose monitoring. In single-parent families only, increased glucose monitoring mediated MST effects on HbA1c.

When MST was compared to telephone support (active control) (Ellis et al., 2012), HbA1c improved, but not long-term (only in adjusted analyses); no reduction in adverse events was found. Parent-reported sustained improvements in diabetes self-care were not confirmed by child reports.

Finally, a pilot study to implement MST in a real-world community setting (“REACH for Control”) suggested improvements in HbA1c (pre: 11.7%, post: 11.0%) compared to care-as-usual (pre: 11.3%, post: 11.4%). Subjective and objective measures of diabetes self-care did not change, while children reported improved quality of life (Ellis et al., 2019).

In summary, MST—targeting the most vulnerable families—appeared primarily successful in improving diabetes self-care and HbA1c in both research and real-world settings, although these effects do not always coincide and appear to be dependent on its measurement, family composition, and trial design. Whereas parental child overestimation decreased overall, effects on other family measures were only observed in two-parent families, and they did not mediate effects of MST on diabetes outcomes. Pilot findings of real-world implementation of MST offer initial support for its effectiveness that should be further explored in a large-scale trial.

### Routine care integrated interventions (8 interventions, 12 studies, 17 reports)

Low-intensity, manualized interventions are designed for parents and/or children to be delivered at quarterly clinic visits over 6–24 months. Most programs are preventive and target all families within specific age bands. They typically aim to prevent reductions in diabetes management by enhancing/maintaining parental involvement without increasing family conflict.

One clinic-integrated intervention provided joint parent–adolescent psychoeducation on constructive communication and teamwork to maintain parental involvement during adolescence (Anderson et al., 1999). Compared to a combined care-as-usual/attention control condition, program participants showed decreases in unsupportive parenting and conflict, while maintaining parental involvement in insulin delivery. The intervention did not affect HbA1c. In contrast, Laffel et al. (2003) did observe an effect on HbA1c (pre: 8.4%, post: 8.2%) compared to care-as-usual (pre: 8.3%, post: 8.7%), while no intervention effects were observed for parental involvement, conflict, or child quality of life.

The above psychoeducational content was incorporated into subsequent programs. First, the combination with a care coordinator (“ambassador”) decreased the annual incidence of severe hypoglycemic events and hospital admissions compared to a combined ambassador-only/care-as-usual control condition (Svoren et al., 2003) and increased parental involvement as compared to ambassador-only (Katz et al., 2014). Neither study showed overall effects on HbA1c, although subgroup analyses suggested benefits for those with elevated baseline HbA1c (≥8.7%). Second, findings of another combination of above-mentioned psycho-education with coping skills training (CST; Holmes et al., 2014) did not favor the program when compared to an educational control condition. The educational control ultimately even showed more improvement over time on self-reported diabetes self-care and HbA1c than the intervention condition, potentially due to its practical care-related focus, as suggested by the authors. Finally, the FACTS intervention combined a similar psychoeducational module with conventional diabetes self-management education. Two studies showed improvements in insulin adjustments to snacks, meals, and glucose levels, without any effects on family-, parent-, or child outcomes or HbA1c compared to care-as-usual (Murphy et al., 2007, 2012).

WE-CAN focused on improving family problem-solving skills to enhance family communication and demonstrated an intervention effect on HbA1c (pre: ∼8.6%, post: ∼8.8%) compared to care-as-usual (pre: ∼8.6%, post: ∼9.4%) among children aged 12–14 years only (Nansel et al., 2012). This effect was accompanied by an (unexplained) decrease in glucose monitoring frequency in the intervention condition. No effects were demonstrated for conflict, parental involvement (Temmen et al., 2022), or hypoglycemic events (Gee et al., 2017). Additional analyses differentiated several parenting classes based on measures of parental involvement and conflict (Lu et al., 2023). Neither these parenting classes nor parental income moderated intervention effects on HbA1c (Nansel et al., 2015; Temmen et al., 2022).

In the single-session “Checking in” intervention, physicians encouraged parents during routine care visits to remain involved by having regular “3-minute-meetings” (Monaghan et al., 2015). An uncontrolled pilot study indicated no improvement in family or diabetes outcomes, yet was not designed or powered to detect changes in these outcomes.

Contrary to the aforementioned programs delivered to all families in certain age bands, the 3MS intervention specifically targeted parents of African American adolescents. An internet-based, animated narrator used motivational interviewing techniques to encourage parents in continued diabetes management supervision. While the intervention showed no overall effects on HbA1c, some effects were found for those with elevated baseline depressive symptoms and diabetes distress (Ellis et al., 2024; Knauft et al., 2024).

Overall, findings for routine-integrated interventions are mixed. Some psychoeducational interventions showed favorable effects on diabetes outcomes but not on family or child outcomes (Laffel et al., 2003; Murphy et al., 2012); others found the opposite pattern (Anderson et al., 1999) or were underpowered to detect impact on outcomes (Monaghan et al., 2015). Effects on diabetes outcomes were found in unexpected directions (Holmes et al., 2014) or were limited to specific age groups (Nansel et al., 2012), or to subgroups with elevated baseline HbA1c (Katz et al., 2014; Svoren et al., 2003) or elevated baseline distress (Knauft et al., 2024). Combinations of different control conditions and multiple intervention components complicated result interpretation.

### Combined parent–child interventions (5 interventions, 7 studies, 7 reports + 2 abstracts)

Regarding parallel parent and child interventions, a trial evaluating Coping Skills Training (CST, addressing coping skills and behavioral patterns) did not find differences in parent, child, diabetes, and most family outcomes compared to a diabetes education condition (Ambrosino et al., 2008; Grey et al., 2009, 2011). Effects on family adaptability (the ability to alter role relationships) were inconsistently reported in the paper (significant improvement in CST vs. control according to some sections, but only trends in other sections). The KIDS intervention combined diabetes education with behavior therapy and family therapy in six parallel parent/adolescent group sessions. After an uncontrolled study showed some preliminary effects (Opipari-Arrigan et al., 2005), a pilot study comparing KIDS to a wait-list control condition did not point to differences in psychosocial or diabetes outcomes (Kichler et al., 2013). On the contrary, effects on child’s quality of life favored the wait-list control condition in this small-scale study, which was suggested to result from selective attrition in this group. An uncontrolled real-world study of KIDS found increases in child readiness for diabetes self-care and a reduction in HbA1c (pre: 9.3%, post: 8.4%, follow-up: 8.6%) (Kichler & Kaugars, 2021).

Two studies examined joint parent–child group sessions. Satin et al. (1989) evaluated two six-session interventions: one where family problem-solving skills were addressed through discussions in multifamily (MF) groups, and one where parents additionally simulated living with diabetes for one week (MF+S). Adolescents in both intervention conditions viewed themselves more positively compared to those receiving care-as-usual. HbA1c values dropped in the MF+S group (pre: 12.6%, −1.21%), but not the MF group (pre: 13.4%, +0.52), compared to care-as-usual (pre: 12.9%, +0.27%); these effects did not sustain. The interventions did not improve family dynamics, diabetes self-care, or other attitudes. Carpenter et al. (2014) evaluated a problem-solving/communication skills training (based on BFST-D components)—delivered as part of clinical care in four group sessions—and showed no significant effects on HbA1c over time.

One additional conference abstract reported insufficient information to evaluate the intervention’s effectiveness (Patel, 2019).

In summary, limited evidence for improvement was found for parallel parent–child trainings compared to group education or care-as-usual, even though interpretation is complicated by small sample sizes. Delivery of a joint MF group was successful in temporarily reducing HbA1c, but only when combined with parental diabetes simulation.

### Stand-alone parent training (10 interventions, 12 studies, 12 reports + 2 conference abstracts)

By focusing on parents as sole agents of parental change, stand-alone parent interventions (generic or diabetes-specific, preventive or targeted) intend to improve child outcomes and ultimately diabetes outcomes.

#### Generic, preventive parent trainings

First, the general, cross-condition Positive Parenting Program (Triple P; 10 hr) teaches parents strategies to address child misbehavior. It was evaluated in varying delivery modes and age bands. A self-directed Triple P intervention for parents of adolescents improved several family and parenting measures compared to care-as-usual, though parental mental health did not change (Doherty et al., 2013). In contrast, parental mental health improved temporarily after professionally delivered Triple P for parents of children 4–12 years compared with care-as-usual. Between-parent conflicts over child-rearing decreased, while HbA1c did not change (Westrupp et al., 2015). Parents of children with preexisting behavior problems reported larger and additional benefits, including short-term effects for child mental health and parenting role satisfaction, delayed effects for ineffective discipline strategies, and sustained effects for diabetes-related conflict and parental mental health. Unexpectedly, Triple P temporarily decreased parental self-efficacy, hypothesized to result from increased parenting self-awareness. Finally, in an uncontrolled study, group-delivered teen Triple P seemed to benefit family, parent, and child outcomes—but not HbA1c—over time (Arkan et al., 2020).

Second, a psychology doctoral candidate provided parents individualized feedback on communication based on an observed parent–adolescent diabetes-related conversation in a single-session intervention (May et al., 2017). In a pilot study, neither adolescents nor parents reported overall increased perceived intimacy and closeness compared to an educational session (only improvement on specific subscales). Person-centered communication indicated improvement, while positive and critical communication did not. As an underpowered pilot study, these results are indicative rather than conclusive.

#### T1D-specific, preventive parent trainings

A 10-hr, group-based parent training for parents of children aged 2–8 years (DELFIN) suggested that HbA1c stabilized (pre: 7.2%, post: 7.1%) compared to an increase in the waitlist control (pre: 7.1%, post: 7.3%) in a feasibility trial (Saßmann et al., 2012). No psychosocial effects were reported post-intervention, while ineffective discipline strategies were decreased at follow-up.

Three less-intensive psychoeducational trainings were evaluated. First, the 4-hr Healthy Living Triple-P training (adapted to chronic conditions, including T1D) showed no benefits for primary caregivers on psychosocial or diabetes outcomes compared to care-as-usual, except for a decrease in corporal punishment (Mitchell et al., 2024). Secondary caregivers (of which few received intervention content) reported beneficial effects for parental self-efficacy, parental adjustment to diabetes, and (parent-reported) child quality of life.

Second, the PRIORITY feasibility trial indicated preliminary evidence of improvements in (pre-)adolescents’ responsiveness to satiety (fullness feelings) after two parental psychoeducational sessions to prevent disordered eating compared to a waitlist control. Although no effects on parent or other child outcomes were found, the study may have been underpowered to draw definite conclusions (Jones et al., 2024).

Third, continuous glucose monitoring (CGM) was combined with five brief psychoeducational sessions for CGM-related behavioral difficulties in young children. Parents reported decreased diabetes burden and fear of hypoglycemia compared to CGM only, although these effects did not sustain. No effects on diabetes outcomes were found [Commissariat et al., 2023; SENCE Study Group, 2021; Van Name et al., 2023].

#### T1D-specific, targeted parent trainings

The T1D-adapted New Authority parent training aims to improve non-coercive parental monitoring in families of adolescents with elevated HbA1c (Rothman-Kabir et al., 2022). Contrary to conventional parenting training (typically focusing on behavioral principles), it relies on four parenting pillars: presence, self-control, support, structure. An uncontrolled pilot study indicated sustained improvement over time in (parental monitoring of) diabetes care, parental helplessness, and average daily glucose levels (12.5, 11.5, and 11.1 mmol/L for pre, post, and follow-up, respectively). Parents, but not children, reported sustained reductions in diabetes conflicts. Objective glucose monitoring did not change.

The diabetes-specific Coping and Communication intervention aims to reduce distress and family conflict by enhancing coping skills and positive parenting skills among distressed mothers (Jaser et al., 2018). When compared to care-as-usual in a pilot study, the intervention improved diabetes conflicts, parental diabetes distress, and children’s quality of life. No changes were found for diabetes outcomes, positive parenting style, parental anxiety, or depressive symptoms, yet a full-scale trial examination should elucidate further effects.

Finally, two conference abstracts point toward intervention effects on HbA1c for a motivational interviewing intervention (pre: 9.0%, post: 8.3%) compared to a waitlist (pre: 9.6%, post: 9.6%) (Kawamura et al., 2012), and a parental authoritativeness intervention (pre: 8.9%, post: 8.2%) compared to diabetes education (pre: 9.1%, post: 9.0%) and care-as-usual (pre: 9.0%, post: 8.9%) (Liberman et al., 2017). However, the absence of full-text publications and methodological details warrants cautious interpretation of these findings.

In conclusion, findings about stand-alone parent trainings were mixed. For adolescents, behavioral principle-based intensive training (e.g., Triple P) suggests most—though no unequivocal—improvements in psychosocial outcomes (Doherty et al., 2013; Jaser et al., 2018). For children ≤12 years, improvements in psychosocial outcomes were most evident for those with preexisting behavioral difficulties (Westrupp et al., 2015), while diabetes outcomes only seem affected after intensive, diabetes-specific parent training (Saßmann et al., 2012). Brief psychoeducational interventions showed short-term (SENCE Study Group, 2021) or limited overall effectiveness (Jones et al., 2024; Mitchell et al., 2024), whereas effects reported by pilot studies should be further examined in studies with larger sample sizes (Jones et al., 2024; May et al., 2017) and controlled designs (Rothman-Kabir et al., 2022).

### Contracting interventions (4 interventions, 4 studies, 5 reports)

Contracting interventions use behavioral contracting (written agreements about target behaviors for child and/or parent, with or without incentives) or related techniques (contingency management/token economy) as primary components to affect child outcomes.

In three interventions (all evaluated in very small-scale, uncontrolled pilot studies), families agreed upon goals for children (regarding diabetes management) and parents (regarding frequency and manner of involvement). Two low-intensity interventions were each delivered in two sessions. The family goal-setting intervention, providing individual and shared reward to parents and children, seemed to improve child-reported diabetes self-care, but no other psychosocial or diabetes outcomes (Halper et al., 2022; Hannon et al., 2018). In a similar intervention (without incentives), families reported improvements in diabetes self-care and HbA1c (pre: 8.1%, post: 7.6%), while psychosocial outcomes did not change (Carroll et al., 2011). However, combining the contract with a “cell phone glucose meter” complicates effect attribution. Extensive contingency management training (14 parallel parent–child sessions) taught parents contingency management skills to increase adolescent’s glucose monitoring alongside individual adolescent therapy (Stanger et al., 2013). Children and parents received monetary incentives for, respectively, increasing glucose monitoring and for rewarding their child accordingly. Pre–post comparisons suggested improvements in self-reported diabetes self-care, daily glucose monitoring frequency (pre: 3.9, post: 6.2), and HbA1c (pre: 11.6%, post: 9.1%), with HbA1c effects measured and sustained at follow-up (9.8%).

Finally, a multiple-baseline study of a token economy-based intervention did not report statistical comparisons (Epstein et al., 1981).

In summary, findings of behavioral contracting as the primary parent intervention focus should be interpreted with caution due to uncontrolled, small-scale pilot designs. Only an intensive, parallel parent–child format while providing monetary incentives suggested some improvements, whereas the effectiveness of short contracting interventions was not convincing or uninterpretable.

### Digital interventions (2 interventions, 2 studies, 2 reports)

Parents completed digitally delivered interventions at home. A pilot feasibility study of the effects of the Type 1 Doing Well app (daily prompts to record and praise their adolescent’s diabetes strengths) did not indicate preliminary effects on (exploratory) outcomes compared to care-as-usual (Hilliard et al., 2020), yet as a pilot feasibility study, the findings should be interpreted with caution given the limited sample size. The Type 1 Teamwork eHealth program (six interactive sessions with information about positive communication, stress management, and responsibility transfer) improved parental mental health but not family or diabetes outcomes compared to a waitlist control (Whittemore et al., 2020).

### Young children (3 interventions, 4 studies, 4 reports)

Interventions for parents of children ≤6 years mainly focus on parental behavioral strategies to change child behavior. Two six-session interventions target child eating behavior (BEST MEALS) or eating behavior and physical activity (Type One Training; TOTs). A pilot RCT to TOTs (focused on parenting skills, e.g., applying consistent routines and managing child behavior alongside peer parent support) offers initial support for improving parental mental health, child eating behavior, and time-in-range (pre: 39.8%, post: 46.2%) compared to care-as-usual (time-in-range pre: 41.0%, post: 43.6%) (Mackey et al., 2022).

An uncontrolled pilot study of BEST MEALS (contingent attention strategies to shape child mealtime behavior) indicated improvement in mean daily glucose levels over time (pre: 10.3 mmol/L, post: 8.8 mmol/L; other outcomes not undergoing statistical testing due to its pilot design) (Patton et al., 2014).

Finally, a pilot study of REDCHiP (10-session, video-based, aiming to reduce parental fear of hypoglycemia and partly addressing parenting skills) suggested pre–post effects on fear of hypoglycemia, but not on other parental mental health outcomes, compared to a waitlist control (Patton et al., 2020). Pre-follow-up comparisons in the intervention condition showed improvement for all parental well-being measures.

In conclusion, controlled pilot trials of interventions targeting young children point toward promising effects on proximal outcome targets (Mackey et al., 2022; Patton et al., 2020), yet warrant large-scale trial confirmation.

### Intervention ingredients

**Figure 1.**
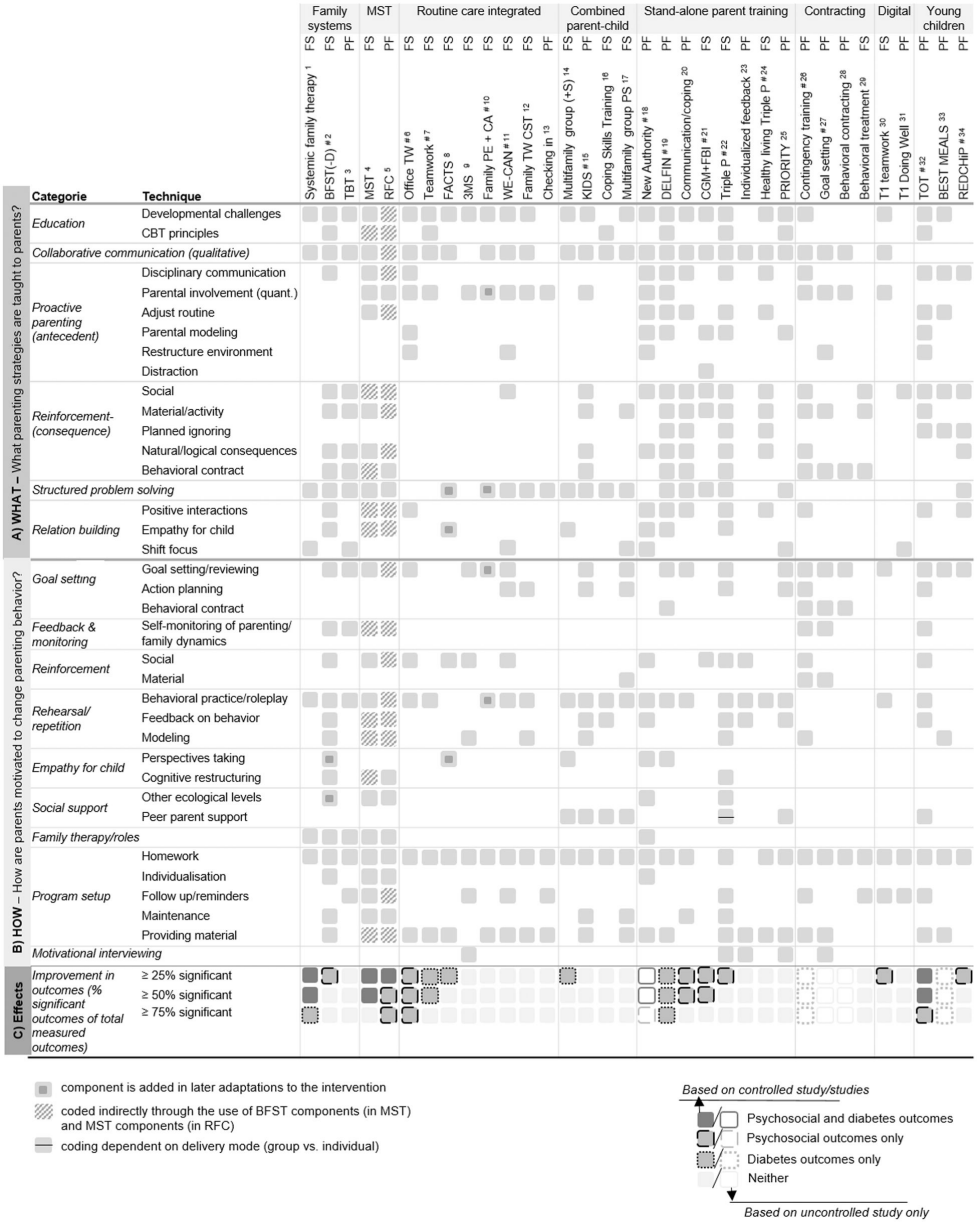
Visual presentation of all components per intervention of those described in full-text papers (*N* = 34 interventions), displaying (A) what parenting strategies are taught to parents, and (B) how parent are motivated to change parenting behavior. Estimated effectiveness (C) is based on total group effects only (i.e., no subgroup effects). ^#^Additional intervention material was provided by authors to code intervention content. FS = findings based on full-scale trial/study; PF = findings based on pilot/feasibility study only; quant. = quantitative (1) Salcudean & Lica (2024); (2) Wysocki et al. (2006, 2007, 2008); (3) Lehmkuhl et al. 2010; (4) Ellis et al. (2005a, 2005b, 2007b, 2007c, 2012), Naar-King et al. (2007); (5) Ellis et al. (2019); (6) Anderson et al. (1999); (7) Laffel et al. 2003; (8) Murphy et al. (2012); (9) Ellis et al. (2024); (10) Katz et al. (2014); (11) Nansel et al. (2012, 2015), Gee et al. (2017), Temmen et al. (2022); (12) Holmes et al. (2014); (13) Monaghan et al. 2015; (14) Satin et al. (1989); (15) Kichler et al. (2013); (16) Ambrosino et al. (2008), Grey et al. (2009, 2011); (17) Carpenter et al. (2014); (18) Rothman-Kabir et al. (2022); (19) Saßmann et al. (2012); (20) Jaser et al. (2018); (21) SENCE Study Group (2021), Commissariat et al. (2023), Van Name et al. (2023); (22) Westrupp et al. (2015), Doherty et al. (2013); (23) May et al. (2017); (24) Mitchell et al. (2024); (25) Jones et al. (2024); (26) Stanger et al. (2013); (27) Halper et al. (2022), Hannon et al. (2018); (28) Carroll et al. (2011); (29) Epstein et al. (1981); (30) Whittemore et al. (2020); (31) Hilliard et al. (2020); (32) Mackey et al. (2022); (33) Patton et al. (2014); (34) Patton et al. (2020).

Regarding *what* parenting strategies were taught, almost all interventions included psychoeducation on developmental challenges and collaborative communication training (Figure 1). At least half of the interventions focused on social rewards (e.g., praise) and problem-solving strategies. BFST, MST, REACH for Control, intensive stand-alone parent trainings, and TOTs each included ≥10 parenting strategies. Regarding *how* parents were stimulated to change, most interventions (≥20) employed goal setting, behavioral practice, or homework/additional material. Few (≤5 interventions) used cognitive restructuring, parental behavioral contracting, individualization, or involvement of ecological levels outside the nuclear family. BFST, MST, Triple-P, TOTs, and contingency training incorporated ≥10 different techniques to motivate parents toward behavioral change.

Intervention effectiveness is displayed using different criteria, based on significant effects/total number of outcomes measured effects percentages (repository includes our decision rules for report selection if multiple studies/reports evaluated an intervention; Jansen et al., 2024).

### Risk of bias assessment

**Figure 2.**
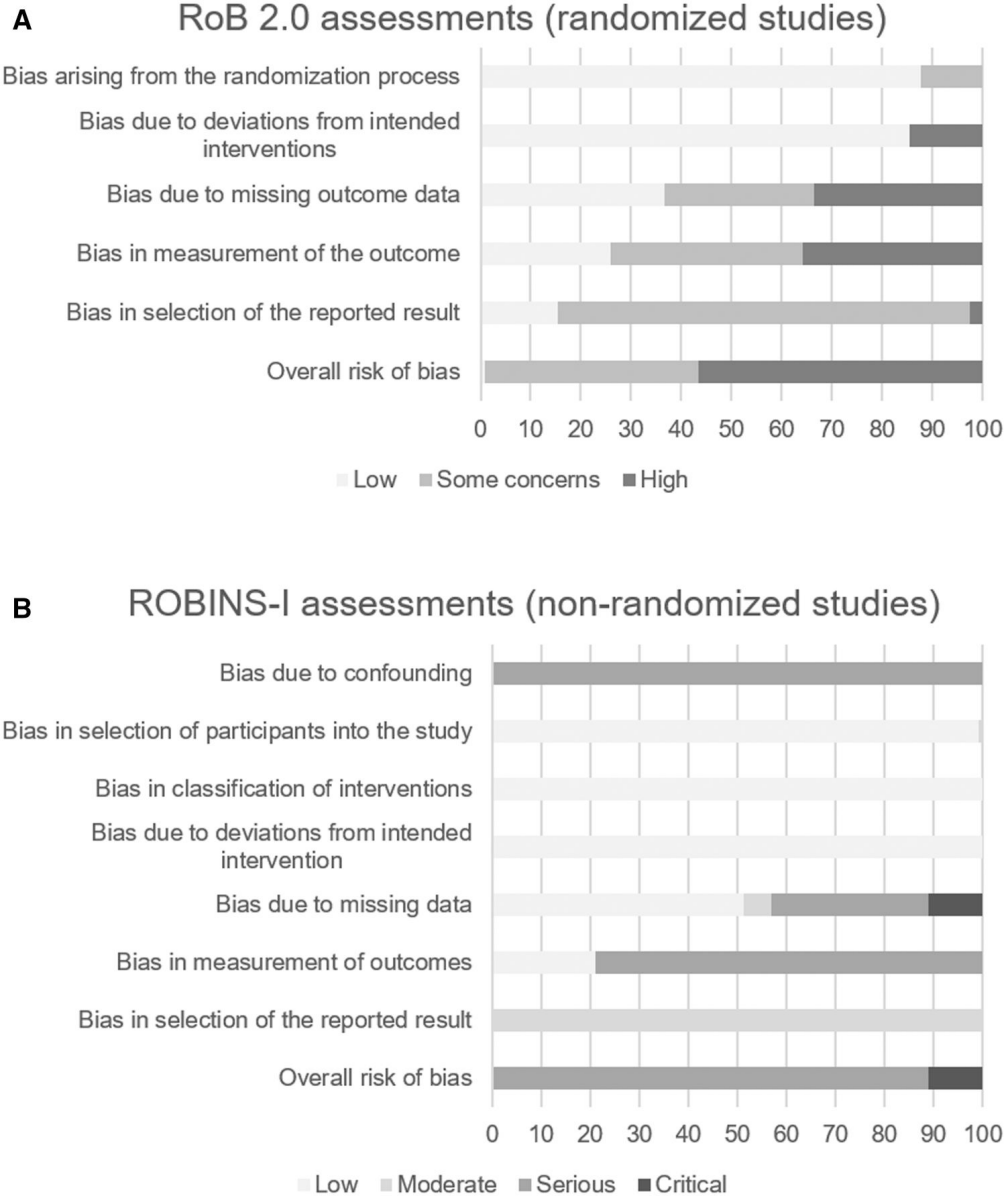
Risk of bias summary plots. Weighted bar plots of the distribution of risk-of-bias judgments overall and within each bias domain, using RoB2.0 tool for randomized-controlled trials (A) and ROBINS-I for non-randomized studies (B).

All 49 full-text RCT reports had more than one outcome category with an increased risk of bias (Figure 2A; Supplementary Figure S2). With some HbA1c outcomes excepted, all outcome categories had their overall risk of bias rated at “some concerns” or “high.” This mostly resulted from study type (e.g., self-reported outcomes) and the absence of pre-specified analysis plans.

We judged all uncontrolled pretest–posttest studies (16 reports) to be at serious or critical risk of bias for ≥1 outcome category (Figure 2B; Supplementary Figure S3). Risk of bias due to confounding was—similar to other reviews (Taylor et al., 2021)—considered serious for all studies across all outcome categories, as it cannot be determined whether changes result from the intervention or extraneous events (Sterne et al., 2024). Bias due to classification of and deviation from intended interventions were mostly judged as low, and bias in the selection of the reported results as moderate. Other domains had variable judgments.

Seven reports did not undergo risk of bias assessment as they were conference abstracts (*N* = 4) or no primary outcome data were extracted (*N* = 3).

## Discussion

This review of 72 reports (51 studies) summarized the effectiveness of 37 parenting interventions in pediatric T1D on various outcomes. Common strategies focused on psycho-education and training in collaborative communication, problem-solving, and praising. Parental change was often stimulated through goal setting, behavioral practice, and homework. Notwithstanding many nonsignificant findings, clearer patterns emerged for certain intervention and outcome types.

First, targeted interventions delivered to clinical or demographic subgroups yielded the most significant findings, although affected outcomes varied. For intensive targeted interventions, MST mostly improved diabetes outcomes with parenting effects limited to demographic subgroups (Ellis et al., 2005a, 2005b, 2007b, 2007c, 2019; Naar-King et al., 2007), while BFST-D improved family communication but affected diabetes outcomes only in clinical subgroups with elevated HbA1c (Wysocki et al., 2006, 2007, 2008). The success of Skype-delivered BFST (Harris et al., 2015) suggests that alternative delivery modes can be explored, although the effects of phone-delivery of a BFST-derived intervention remain to be elucidated in a large-scale trial (Lehmkuhl et al., 2010). Similarly, the promising initial findings of the New Authority intervention (Rothman-Kabir et al., 2022) and systemic family psychotherapy (Salcudean & Lica, 2024) warrant further evaluation. For briefer-targeted interventions, addressing coping/communication among mothers with elevated distress seemed to reduce conflicts and distress (Jaser et al., 2018), while targeting monitoring among African-American parents only improved diabetes outcomes among subgroups of children with elevated distress or depressive symptoms (Ellis et al., 2024; Knauft et al., 2024). In conclusion, targeted interventions—particularly MST and BFST-D—are able to impact family and diabetes outcomes, though even within these target populations, many effects appear limited to specific demographic (e.g., two-parent households) and clinical subgroups (e.g., elevated baseline HbA1c or distress).

Second, preventive interventions for all families in certain age bands had mixed effects. Stand-alone parent trainings based on behavioral principles (e.g., Triple P) were generally most effective, particularly for families with preexisting difficulties (Doherty et al., 2013; Jaser et al., 2018; Saßmann et al., 2012; Westrupp et al., 2015). For other intervention formats (routine care integrated and combined parent–child interventions), only a few interventions pointed toward overall benefits among many nonsignificant findings (Anderson et al., 1999; Laffel et al., 2003; Murphy et al., 2012; Satin et al., 1989), or showed effects in specific age groups only (Nansel et al., 2012). These interventions all varied greatly in content and affected domains. Notably, many studies reporting null effects were small pilot trials likely underpowered to detect meaningful differences. As to specific techniques, behavioral contracting embedded in other interventions showed some beneficial effects (Ellis et al., 2005a; Jaser et al., 2018; Saßmann et al., 2012; Stanger et al., 2013; Wysocki et al., 2006), whereas effects of contracting as the intervention’s sole ingredient warrant further investigation (Carroll et al., 2011; Hannon et al., 2018). Similarly, promising interventions focusing on young children (Mackey et al., 2022; Patton et al., 2020) or eHealth (Whittemore et al., 2020) need trial confirmation. In summary, our findings suggest that the effects of universally delivered preventive programs are less robust and tend to benefit specific subgroups, though further research is needed before drawing firm conclusions, particularly given the high number of small-scale pilot studies in this area.

As to outcomes, changes in psychosocial and diabetes measures often did not align. Only a few studies found effects on diabetes outcomes, which is in accordance with previous meta-analyses that did not find an overall effect on diabetes outcomes (Law et al., 2019; Winkley et al., 2020). Interventions that did improve diabetes outcomes were all diabetes-specific and either more intensive (Ellis et al., 2007b; Rothman-Kabir et al., 2022; Salcudean & Lica, 2024; Saßmann et al., 2012; Wysocki et al., 2006, 2007), aimed at young children (Mackey et al., 2022; Patton et al., 2014), practical and/or education-focused (Ellis et al., 2024; Holmes et al., 2014; Laffel et al., 2003; Murphy et al., 2012; Nansel et al., 2012; Svoren et al., 2003), or included parental diabetes-simulation (McBroom & Enriquez, 2009; Satin et al., 1989; Wysocki et al., 2006). Inconsistencies in HbA1c effects of data from the same trial could possibly be explained by different analyses and imputation strategies, as one analysis yielded significant effects (Ellis et al., 2007b) as compared to two reports—both using different analysis strategies—describing trend effects (Ellis et al., 2005a, 2007c). These inconsistencies raise concerns about the robustness of effects. HbA1c effects could result from a greater decrease in the intervention vs. control condition (Ellis et al., 2007b, 2012; Satin et al., 1989) or from HbA1c stabilization in the intervention vs. an increase in the control condition (Laffel et al., 2003; Saßmann et al., 2012). Interestingly, parenting outcomes did not always improve concomitantly with diabetes outcomes (Holmes et al., 2014; Laffel et al., 2003; Nansel et al., 2012; Saßmann et al., 2012) or only in subgroups (Ellis et al., 2007c). Effects on parenting outcomes were mostly evident for diabetes-specific measures of parental involvement (Katz et al., 2014; Kichler & Kaugars, 2021; Naar-King et al., 2007; Rothman-Kabir et al., 2022) and/or conflict (Anderson et al., 1999; Arkan et al., 2020; Doherty et al., 2013; Harris & Mertlich, 2003; Rothman-Kabir et al., 2022; Wysocki et al., 2006), but less often for general parenting measures (Ellis et al., 2007c; Jaser et al., 2018; May et al., 2017; Mitchell et al., 2024; Saßmann et al., 2012; Westrupp et al., 2015; Whittemore et al., 2020; Wysocki et al., 2006). The large heterogeneity in intervention content and selected outcomes precludes statements regarding essential intervention components for affecting parenting outcomes. Summarizing, interventions impacting HbA1c are scarce yet share some commonalities (diabetes-specific, intensive, practical, aimed at young children, and/or including diabetes simulation); effects on diabetes-specific parenting outcomes are reported more often, yet also more scattered and not relatable to unique features of interventions.

The discordance between psychosocial and diabetes outcomes questions whether psychosocial effects should be considered as intervention endpoints, mechanisms, or both. While improved family interactions are an endpoint from a psychological perspective, they are considered a mechanism to improve diabetes outcomes from a medical perspective. The latter presumption was, however, not always supported (Ellis et al., 2007c; Grey et al., 2009). Possibly, interventions directly affected diabetes outcomes (Ellis et al., 2007c), or generic parenting measures did not capture changes in diabetes-specific parenting (Wysocki et al., 2006). Our results suggest that for proper intervention evaluation, psychosocial endpoints need to be considered alongside medical criteria such as HbA1c (Feldman et al., 2018; Hampson et al., 2000), using appropriate and updated diabetes-specific measures of family behavior.

Our review has important implications for both research and clinical care regarding allocating resources to preventive and/or targeted approaches in T1D. Concerning implications for targeted interventions, our findings suggest that—in line with other literature—flexible, multisystemic, and outreaching interventions are warranted for the most vulnerable (e.g., “high-need, high-cost”) families (Barry-Menkhaus et al., 2020; Wagner et al., 2015). Intensive interventions such as MST are resourceful yet cost-effective by reducing hospitalizations (Ellis et al., 2008a; Harris et al., 2014) and show promising outcomes when implemented in the real-world setting (Ellis et al., 2019). Similarly, children enrolled in the intensive home- and community-based Novel Interventions in Children’s Healthcare (NICH) show preliminary positive outcomes for improving health and care, and reducing costs during program involvement (Harris et al., 2014, 2016; Wagner et al., 2015, 2017). Absence of post-intervention (instead of post-enrollment) outcome data precluded inclusion of NICH in the current review, which should be further explored (Ellis et al., 2019). Challenges for research and clinical practice for these intensive interventions particularly concern strategies to increase implementation and reach of these programs. For implementation, lessons can be learned from studies that successfully implemented these programs, which include seeking sustainable collaborations with local community workers (Ellis et al., 2019). For enhancing reach, we suggest exploring online/remote delivery, as some studies point to similar effectiveness as when delivered in clinic (Delamater & Marrero, 2020; Harris et al., 2015).

Regarding implications for preventive interventions, our findings should be interpreted in light of the nature of these interventions. Some of our findings question the utility of preventive interventions, as several effects were equal to or even smaller than active control conditions (Ambrosino et al., 2008; Grey et al., 2011; Holmes et al., 2014), temporarily negative (Holmes et al., 2014; Lehmkuhl et al., 2010; Westrupp et al., 2015), or limited to/more pronounced in clinical subgroups (Ellis et al., 2024; Katz et al., 2014; Knauft et al., 2024; Svoren et al., 2003; Westrupp et al., 2015). Preventive interventions, nevertheless, should not be discarded immediately for several reasons. First, several studies that did demonstrate preventive intervention effects involved larger sample sizes, indicating that some (pilot) studies with null findings may have been underpowered to detect meaningful effects. Second, preventive effects are generally difficult to demonstrate since participants have low base rates of difficulties (Mitchell et al., 2024) and distal effects require long-term follow-up (Grey et al., 2009). Third, educational interventions enhanced with family therapy generally show more promising effects than education per se (Savage et al., 2010). Finally, preventive (routine-integrated) interventions generally have a larger reach—even though recruitment/attendance can be challenging (Carpenter et al., 2014; Murphy et al., 2007, 2012), which has also been reported for other chronic conditions (Morawska et al., 2015). Therefore, the next steps are to elucidate potential distal preventive effects and identify who benefits from preventive parenting interventions. Besides, strategies to enhance reach and recruitment could be explored, although one should carefully consider whether all parents of chronically ill children in fact want to dedicate time and energy to parenting support, especially in light of already high disease burden and potential low perceived need (Mitchell et al., 2020; Morawska et al., 2015).

Considering our review findings alongside these general matters regarding preventive interventions, we recommend assessing for individual families whether they would benefit from a (preventive) parenting program vs. another high-quality (educational) intervention (Hilliard et al., 2016). An attempt to tailor a preventive intervention to familys’ needs—including sessions on family teamwork, education, and motivational interviewing—showed preliminary effects, albeit also most pronounced in those with initial suboptimal HbA1c levels (Fiallo-Scharer et al., 2019). Additionally, a cross-condition approach to parenting interventions has been suggested to enhance intervention sustainability, as many chronic diseases share common difficulties (Mitchell et al., 2020; Morawska et al., 2015). However, as our review and others (Hood & Nansel, 2007; Wysocki et al., 2006) suggest that augmentation with diabetes-specific elements increases intervention effects, we advocate for careful consideration of incorporating illness-specific elements if adopting cross-condition approaches.

This review has several strengths as compared to existing reviews related to this topic. First, it provides a comprehensive overview of all available literature in this field by also including gray literature and pilot/feasibility studies. Although these studies provide less robust evidence than large-scale trials, they offer researchers insight into current developments and a foundation for identifying gaps and priorities for future studies. Second, by systematically coding the active components of each intervention and grouping them based on their interrelatedness, we move beyond a generic summary of programs to offer insights into the specific elements being applied. This approach helps to unpack the “black box” of intervention content and can guide practitioners in selecting or adapting strategies that best match their context, resources, and goals. Methodological limitations of our review include the moderate/high risk of bias assessments—inherent to most self-reported outcomes and/or non-blinded studies—and low sample sizes (*N* ≤ 40) for many (pilot) studies (Arkan et al., 2020; Carroll et al., 2011; Epstein et al., 1981; Hannon et al., 2018; Harris & Mertlich, 2003; Jaser et al., 2018; Kichler & Kaugars, 2021; Kichler et al., 2013; Mackey et al., 2022; Monaghan et al., 2015; Patton et al., 2014; Rothman-Kabir et al., 2022; Salcudean & Lica, 2024; Saßmann et al., 2012; Satin et al., 1989; Stanger et al., 2013; Tully et al., 2018). Additionally, limited diversity regarding the socioeconomic and racial background of samples limits the generalizability of findings, as people from minority groups, on average, experience more adverse diabetes outcomes (Lipman et al., 2021) and lower levels of parental monitoring (Ellis et al., 2008b), and may warrant unique approaches. Furthermore, while our prioritization of intention-to-treat analyses on overall group effects increased review rigor, it did exclude intervention effects based on uncontrolled pre–post differences in controlled study designs (Commissariat et al., 2023; Kichler et al., 2013; Patton et al., 2020; Saßmann et al., 2012) or from exploratory analyses, such as effects restricted to attendees (Carpenter et al., 2014; Murphy et al., 2007, 2012), to certain study cycles (Satin et al., 1989), or to parents who implemented taught strategies at home (Monaghan et al., 2015). This may also explain why our overall conclusions might appear somewhat less positive as compared to some related reviews (Feldman et al., 2018; Hilliard et al., 2016; McBroom & Enriquez, 2009). Finally, given the large heterogeneity in extracted outcomes, we prioritized outcome-specific quality assessments of individual studies over overall certainty of the evidence, which can be considered a limitation.

In conclusion, this systematic review found mixed effects of parenting interventions in pediatric T1D. Intensive interventions targeting families with existing difficulties appear most effective, although some preventive interventions and educational control groups also showed effects and warrant further investigation. This underlines the importance of matching intervention allocation to the target group. A diabetes-specific focus seems necessary, although not sufficient, for impacting diabetes outcomes. Future high-quality trials and real-world studies using diabetes-specific medical and family endpoints should elucidate which families benefit from parenting intervention (elements).

## Supplementary Material

jsaf078_Supplementary_Data

## Data Availability

Most of the data underlying this article are available in the article, its online supplementary material, and in Open Science Framework (OSF) (Jansen et al., 2024). Part of the data underlying this article were provided by authors who shared intervention manuals under license and by permission. These data will be shared on request after permission from the respective authors is obtained.
