# Supplementary material for: Parenting interventions for parents of children with type 1 diabetes—a systematic review
Source: J Pediatr Psychol. 2025 Sep 22;50(12):1115–38. doi: 10.1093/jpepsy/jsaf078 (PMC12755088; doi:10.1093/jpepsy/jsaf078)
Supplement: jsaf078_Supplementary_Data [file jsaf078_supplementary_data.zip › jsaf078_Supplementary_Data/jpepsy-2024-0314-File008_final.docx]

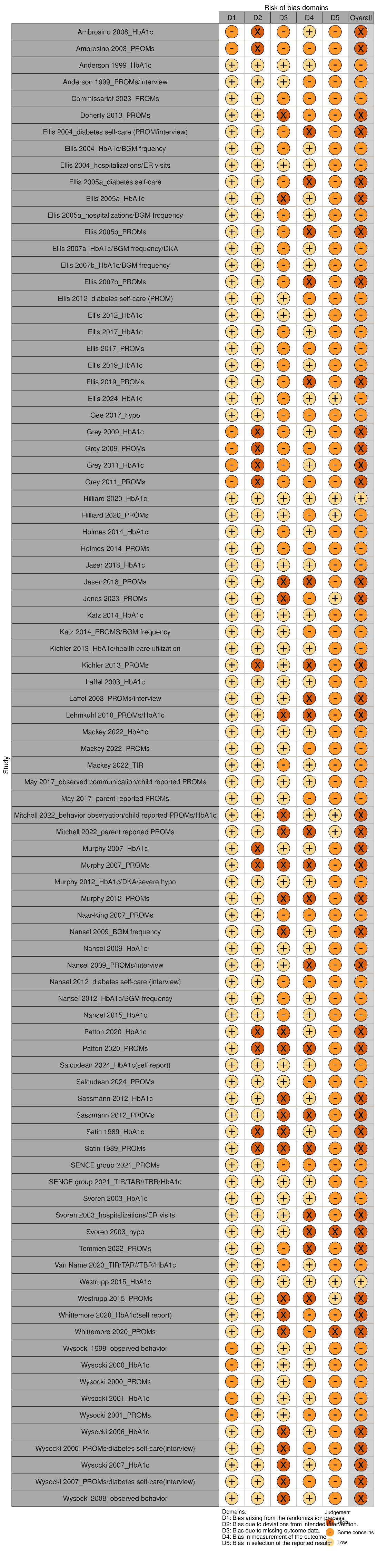

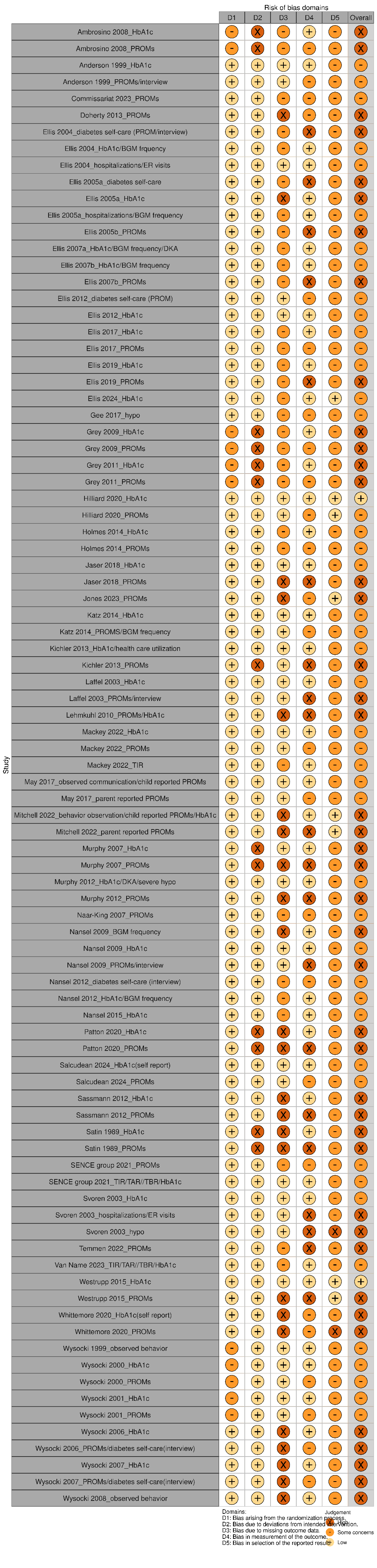


**Supplementary Figure S2:** Risk of bias traffic plots for all outcomes of included studies that have been assessed using the risk-of-bias (RoB) 2.0 tool.

Each outcome and time point is assessed separately. For parsimonious reasons, outcomes within one outcome category (e.g. PROMs) are collated as they usually yielded the same risk of bias (RoB) judgement, and outcome categories yielding identical RoB assessments are displayed in one line. If RoB assessments differed for outcomes within one outcomes category or for different time points (e.g. post/follow up), they are reported separately; if no time point is specified, both extracted time points (if applicable) yielded the same RoB assessment.

PROMs = person reported outcome measures; BGM = blood glucose monitoring; ER = emergency room; hypo = hypoglycemia; TIR = time-in-range; DKA = diabetic ketoacidosis; TAR = time-above-range; TBR = time-below-range

Reports of Lu, 2023 and Knauft, 2024 did not undergo RoB assessment, as no new primary outcomes were reported (only additional analyses); reports of Kawamura, 2012 and Liberman, 2017 did not undergo RoB assessment as they concern conference abstracts; report of Epstein, 1989 did not undergo RoB assessment as no data were extracted.

**Alt text:** Risk of bias traffic plots for all outcomes of included studies that have been assessed using the ROBINS-I tool. The plots use different symbols to represent the level of risk of bias (low, some concerns, high) for each outcome per study. They provide an overview of the overall risk of bias, as well as for each individual domain (bias due to randomization, deviations from intended interventions, missing outcome data, measurement of outcomes, and selective reporting).
