# Supplementary material for: Parenting interventions for parents of children with type 1 diabetes—a systematic review
Source: J Pediatr Psychol. 2025 Sep 22;50(12):1115–38. doi: 10.1093/jpepsy/jsaf078 (PMC12755088; doi:10.1093/jpepsy/jsaf078)
Supplement: jsaf078_Supplementary_Data [file jsaf078_supplementary_data.zip › jsaf078_Supplementary_Data/jpepsy-2024-0314-File009_final.docx]

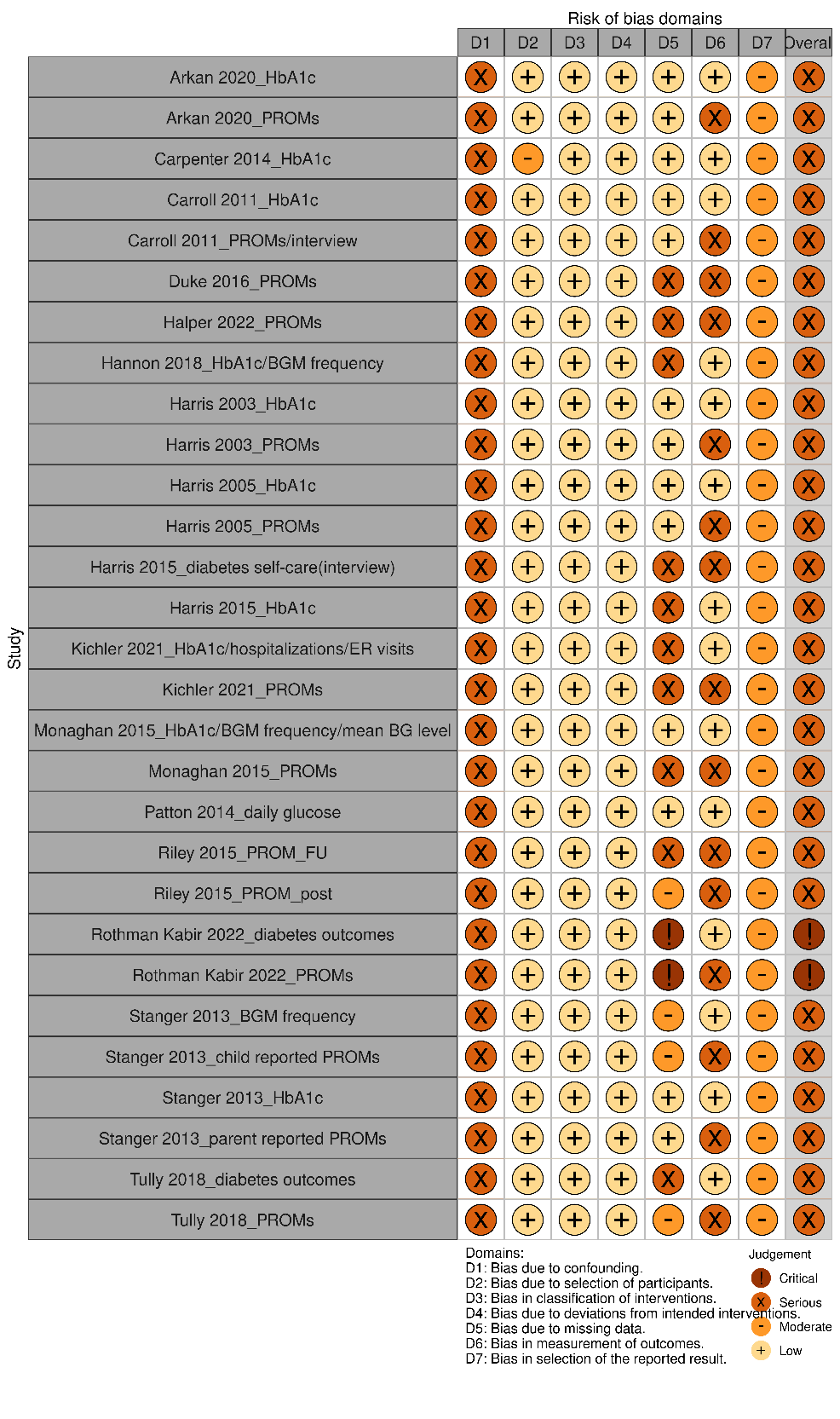


**Supplementary Figure S3:** Risk of bias traffic plots for all outcomes of included studies that have been assessed using the ROBINS-I tool.

Each outcome and time point is assessed separately. For parsimonious reasons, outcomes within one outcome category (e.g. PROMs) are collated as they usually yielded the same risk of bias (RoB) judgement, and outcome categories yielding identical RoB assessments are displayed in one line. If RoB assessments differed for outcomes within one outcomes category or for different time points (e.g. post/follow up), they are reported separately; if no time point is specified, both extracted time points (if applicable) yielded the same RoB assessment.

PROMs = person reported outcome measures; BGM = blood glucose monitoring; ER = emergency room; BG = blood glucose; FU = follow up

Reports of Opipari, 2005 and Patel, 2019 did not undergo RoB assessment as they concern conference abstracts

**Alt text**: Risk of bias traffic plots for all outcomes of included studies that have been assessed using the ROBINS-I tool. The plots use different symbols to represent the level of risk of bias (low, serious, moderate, high) for each outcome per study. They provide an overview of the overall risk of bias, as well as for each individual domain (bias due to confounding, selection of participants, classification of interventions, deviation from intended interventions, missing data, measurement of outcomes, and selective reporting).
