# Supplementary material for: Parenting interventions for parents of children with type 1 diabetes—a systematic review
Source: J Pediatr Psychol. 2025 Sep 22;50(12):1115–38. doi: 10.1093/jpepsy/jsaf078 (PMC12755088; doi:10.1093/jpepsy/jsaf078)
Supplement: jsaf078_Supplementary_Data [file jsaf078_supplementary_data.zip › jsaf078_Supplementary_Data/jpepsy-2024-0314-File010_final.docx]

**Supplementary file S2** – Full search strategy

Initial search: September 9th, 2022

Updated search: January 17^th^, 2024

Second updated search: February 25^th^, 2025

# PubMed

## #1 Diabetes type 1

(diabetes mellitus, type 1[MesH] OR “diabetes mellitus type 1” [TIAB] OR “diabetes mellitus type I” [TIAB] OR “diabetes type 1” [TIAB] OR “diabetes type I” [TIAB] OR “type 1 diabetes” [TIAB] OR T1D [TIAB] OR T1DM [TIAB] OR “insulin-dependent diabetes” [TIAB] OR “juvenile diabetes” [TIAB] OR “juvenile-onset diabetes” [TIAB] OR “sudden-onset diabetes mellitus” [TIAB] OR “autoimmune diabetes” [TIAB] OR IDDM [tiab])

## #2 Children

(adolescent[MesH] OR child[MesH] OR Infant [MesH] OR pediatrics [MesH] OR child* [TIAB] OR adolesc* [TIAB] OR infan* [TIAB] OR baby [TIAB] OR babies [TIAB] OR toddler* [TIAB] OR teen* [TIAB] OR youth* [TIAB] OR pediat* [TIAB] OR paediat* [TIAB] OR kid [TIAB] OR kids [TIAB] OR girl* [TIAB] OR boy [TIAB] OR boys [TIAB] OR juvenile* [TIAB] )

## #3 Parents

(Family [MesH] OR Caregivers[Mesh] OR parent* [TIAB] OR caregiv* [TIAB] OR care-giv* [TIAB] OR father* [TIAB] OR mother* [TIAB] OR maternal [TIAB] OR paternal [TIAB] OR family [TIAB] OR families [TIAB])

## #4 Intervention / psychotherapy

(Psychotherapy[Mesh] OR Health education [MesH] OR Education, Nonprofessional [MesH] OR Program Evaluation [MesH] OR Treatment Outcome [MesH] OR interven* [TIAB] OR program* [TIAB] OR support* [TIAB] OR training* [TIAB] OR course* [TIAB] OR educat* [TIAB] OR therap* [TIAB] OR treatment* [TIAB] OR psychotherap* [TIAB])

**#1 AND #2 AND #3 AND #4**

# Embase

## #1 Diabetes type 1

(exp insulin dependent diabetes mellitus/ OR ("diabetes mellitus type 1" OR "diabetes mellitus type I" OR "diabetes type 1" OR "diabetes type I" OR "type 1 diabetes" OR T1D OR T1DM OR "insulin-dependent diabetes" OR "juvenile diabetes" OR "juvenile-onset diabetes" OR "sudden-onset diabetes mellitus" OR "autoimmune diabetes" OR IDDM ).ti,ab,kf.)

## #2 Children

(exp adolescent/ OR exp adolescence/ OR exp child/ OR exp childhood disease/ OR exp infant disease/ OR exp adolescent disease/ OR exp pediatrics OR (child* OR adolesc* OR infan* OR baby OR babies OR toddler* OR teen* OR youth* OR pediat* OR paediat* OR kid OR kids OR girl* OR boy OR boys OR juvenile*).ti,ab,kf.)

## #3 Parents

(exp family/ OR exp Caregiver/ OR (parent* OR caregiv* OR care-giv* OR father* OR mother* OR maternal OR paternal OR family OR families).ti,ab,kf.)

## #4 Intervention / psychotherapy

(exp Psychotherapy/ OR exp health education/ OR exp program evaluation/ OR exp treatment outcome/ OR (interven* OR program* OR support* OR training* OR course* OR educat* OR therap* OR treatment* OR psychotherap*).ti,ab,kf.)

**#1 AND #2 AND #3 AND #4**

# PsycInfo

## #1 Diabetes type 1

(exp diabetes mellitus/ OR ("diabetes mellitus type 1" OR "diabetes mellitus type I" OR "diabetes type 1" OR "diabetes type I" OR "type 1 diabetes" OR T1D OR T1DM OR "insulin-dependent diabetes" OR "juvenile diabetes" OR "juvenile-onset diabetes" OR "sudden-onset diabetes mellitus" OR "autoimmune diabetes" OR IDDM ).ti,ab,id.)

## #2 Children – searchblock BMI?

(childhood birth 12 yrs OR adolescence 13 17 yrs).ag. OR exp pediatrics/ OR chronically ill children/ OR (child* OR adolesc* OR infan* OR baby OR babies OR toddler* OR teen* OR youth* OR pediat* OR paediat* OR kid OR kids OR girl* OR boy OR boys OR juvenile*).ti,ab,id.

## #3 Parents

(exp family/ OR exp Caregivers/ OR (parent* OR caregiv* OR care-giv* OR father* OR mother* OR maternal OR paternal OR family OR families).ti,ab,id.)

## #4 Intervention / psychotherapy

(exp Psychotherapy/ OR exp client education/ OR exp psychoeducation/ OR exp family life education/ OR exp program evaluation/ OR exp treatment outcomes/ OR (interven* OR program* OR support* OR training* OR course* OR educat* OR therap* OR treatment* OR psychotherap*).ti,ab,id.)

**#1 AND #2 AND #3 AND #4**

## CINAHL

## #1 Diabetes type 1

(MH "Diabetes Mellitus, Type 1+") OR TI ("diabetes mellitus type 1" OR "diabetes mellitus type I" OR "diabetes type 1" OR "diabetes type I" OR "type 1 diabetes" OR T1D OR T1DM OR "insulin-dependent diabetes" OR "juvenile diabetes" OR "juvenile-onset diabetes" OR "sudden-onset diabetes mellitus" OR "autoimmune diabetes" OR IDDM ) OR AB ("diabetes mellitus type 1" OR "diabetes mellitus type I" OR "diabetes type 1" OR "diabetes type I" OR "type 1 diabetes" OR T1D OR T1DM OR "insulin-dependent diabetes" OR "juvenile diabetes" OR "juvenile-onset diabetes" OR "sudden-onset diabetes mellitus" OR "autoimmune diabetes" OR IDDM)

## #2 Children – searchblock BMI?

(MH "Child+") OR (MH "Adolescence+") OR (MH "Pediatrics+") OR TI (child* OR adolesc* OR infan* OR baby OR babies OR toddler* OR teen* OR youth* OR pediat* OR paediat* OR kid OR kids OR girl* OR boy OR boys OR juvenile*) OR AB (child* OR adolesc* OR infan* OR baby OR babies OR toddler* OR teen* OR youth* OR pediat* OR paediat* OR kid OR kids OR girl* OR boy OR boys OR juvenile*)

## #3 Parents

(MH "Family+")OR (MH "Caregivers+") OR TI (parent* OR caregiv* OR care-giv* OR father* OR mother* OR maternal OR paternal OR family OR families) OR AB (parent* OR caregiv* OR care-giv* OR father* OR mother* OR maternal OR paternal OR family OR families)

## #4 Intervention / psychotherapy

(MH "Psychotherapy+")OR (MH "Health Education+") OR (MH “Program Evaluation”) OR (MH “Treatment Outcomes+”) OR TI (interven* OR program* OR support* OR training* OR course* OR educat* OR therap* OR treatment* OR psychotherap*) OR AB (interven* OR program* OR support* OR training* OR course* OR educat* OR therap* OR treatment* OR psychotherap*)

**#1 AND #2 AND # AND #4**

# Cochrane

## #1 Diabetes type 1

("diabetes mellitus type 1" OR "diabetes mellitus type I" OR "diabetes type 1" OR "diabetes type I" OR "type 1 diabetes" OR T1D OR T1DM OR "insulin-dependent diabetes" OR "juvenile diabetes" OR "juvenile-onset diabetes" OR "sudden-onset diabetes mellitus" OR "autoimmune diabetes" OR IDDM ):ti,ab,kw

## #2 Children

(child* OR adolesc* OR infan* OR baby OR babies OR toddler* OR teen* OR youth* OR pediat* OR paediat* OR kid OR kids OR girl* OR boy OR boys OR juvenile*):ti,ab,kw

## #3 Parents

(parent* OR caregiv* OR care-giv* OR father* OR mother* OR maternal OR paternal OR family OR families):ti,ab,kw

## #4 Intervention / psychotherapy

(interven* OR program* OR support* OR training* OR course* OR educat* OR therap* OR treatment* OR psychotherap*):ti,ab,kw

**#1 AND #2 AND #3 AND #4**
