# Supplementary material for: Parenting interventions for parents of children with type 1 diabetes—a systematic review
Source: J Pediatr Psychol. 2025 Sep 22;50(12):1115–38. doi: 10.1093/jpepsy/jsaf078 (PMC12755088; doi:10.1093/jpepsy/jsaf078)
Supplement: jsaf078_Supplementary_Data [file jsaf078_supplementary_data.zip › jsaf078_Supplementary_Data/jpepsy-2024-0314-File011_final.docx]

| Supplementary Table S1 – Extracted data of included studies on post and first follow up measures | | | | | | | | | | | |
| --- | --- | --- | --- | --- | --- | --- | --- | --- | --- | --- | --- |
| Reports (first author, year) | Intervention, control | Reported effects (analyses, time-points, N) | Outcome (measure) (modality) - *(subscale)* | Parenting/  Family dynamics | | Parent | | Child | | Diabetes | |
|  |  |  |  | Post | FU | Post | FU | Post | FU | Post | FU |
| **Family Systems Therapy** | | | | | | | | | | | |
| Wysocki, 1999;  Wysocki, 2000;  Wysocki, 2001 | Behavioral Family Systems Therapy (BFST),  educational control (EC), CAU | initial group*time RM-ANOVA and ANCOVA results were N.S.;  reported results are:  ^$^ANCOVA on change score, group effect, adjusted;  ^$$^MANCOVA on post & FU values, group effect, adjusted  Pre, post, 9 mo. FU;  (N=78) | Parent-child relationship (PARQ) (mean PR+CR) – *overt conflict/skill deficits* | EC: N.S.^$^  CAU: p<.03^$^ | EC: N.S.^$^  CAU: p<.05^$^ |  |  |  |  |  |  |
|  |  |  | Parent-child relationship (PARQ) (mean PR+CR) – *family structure* | EC/CAU: both N.S.^$^ | EC/CAU: both N.S.^$^ |  |  |  |  |  |  |
|  |  |  | Parent-child relationship (PARQ) (mean PR+CR) – *extreme beliefs** | EC/CAU: both sign (p NR) ^$^ | EC/CAU: both p<.05^$^ |  |  |  |  |  |  |
|  |  |  | Conflicts (IC) (mean PR+CR) – *no. of conflict items** | EC/CAU: both sign (p NR) ^$^ |  |  |  |  |  |  |  |
|  |  |  | Conflicts (IC) (mean PR+CR) – *frequency* | EC/CAU: both N.S. ^$^ |  |  |  |  |  |  |  |
|  |  |  | Conflicts (IC) (mean PR+CR) – *intensity* | EC/CAU: both sign (p NR) ^$^ |  |  |  |  |  |  |  |
|  |  |  | Diabetes conflict (DRCS) (mean PR+CR) – *conflict* | EC: p<.05^$^  CAU: p<.04^$^ | EC: p<.05^$^  CAU: p<.04^$^ |  |  |  |  |  |  |
|  |  |  | Conflict (recall interview) (I – mean PR+CRI) – *3 subscales* | EC: all N.S.^$^  CAU: all N.S.^$^ |  |  |  |  |  |  |  |
|  |  |  | Negative reciprocity (IBC) (OR) | group p<.05^$$^ (contrasts NR) | |  |  |  |  |  |  |
|  |  |  | Positive reciprocity (IBC) (OR) | group: N.S. ^$$^ (contrasts NR) | |  |  |  |  |  |  |
|  |  |  | Problem-solving process (IBC) (OR) | group: N.S. ^$$^ (contrasts NR) | |  |  |  |  |  |  |
|  |  |  | Problem resolution (IBC) (OR) | group: p<.03^$$^ (contrasts NR) | |  |  |  |  |  |  |
|  |  |  | Negative communication (IBC) (OR) - *mother* | EC/CAU: both sign (p<.05) ^$$^ | EC/CAU: both sign (p<.05) ^$$^ |  |  |  |  |  |  |
|  |  |  | Negative communication (IBC) (OR) - *father* | EC/CAU: both N.S. ^$$^ | |  |  |  |  |  |  |
|  |  |  | Positive communication (IBC) (OR) – *mother&father&child* | EC/CAU: all N.S. ^$$^ | |  |  | EC/CAU: both N.S. ^$$^ | |  |  |
|  |  |  | Negative communication (IBC) (OR) - *child* |  |  |  |  | EC: N.S.^$$^  CAU: sign (p <.05)^$$^ | EC: N.S. ^$$^  CAU: sign (p <.05)^$$^ |  |  |
|  |  |  | Adjustment to T1D (TADS) (mean PR+ CR) |  |  |  |  | EC/CAU: both N.S. ^$^ | EC/CAU: both N.S. ^$^ |  |  |
|  |  |  | Diabetes self-care (24 hour recall) (I – mean PR+CR) - 5 scores |  |  |  |  |  |  | EC/CAU: all N.S. ^$^ |  |
|  |  |  | Long-term diabetes care (SCI) (mean PR+CR) |  |  |  |  |  |  | EC/CAU: both N.S. ^$^ | EC/CAU: both p<.05^$^ |
|  |  |  | Hospitalizations (OM) |  |  |  |  |  |  | EC/CAU: both N.S. ^$^ | EC/CAU: both N.S. ^$^ |
|  |  |  | Emergency room visits (OM) |  |  |  |  |  |  | EC/CAU: both N.S. ^$^ | EC/CAU: both N.S. ^$^ |
|  |  |  | HbA1c (OM) |  |  |  |  |  |  | EC/CAU: both N.S. ^$^ | EC/CAU: both N.S. ^$^ |
| Supplementary Table 1 – Extracted data of included studies on post and first follow up measures *(continued – 2)* | | | | | | | | | | | |
| Reports (first author, year) | Intervention, control | Reported effects (analyses, time-points, N) | Outcome (measure) (modality) - *(subscale)* | Parenting/  Family dynamics | | Parent | | Child | | Diabetes | |
|  |  |  |  | Post | FU | Post | FU | Post | FU | Post | FU |
| Harris 2003, Harris 2005 | BFST  (home),  no control | Paired t-tests  Pre, post, ~8 mo. FU;  (N=18)  Pilot | Diabetes conflict (DRCS) (MR) | p=.002 | N.S. |  |  |  |  |  |  |
|  |  |  | Diabetes conflict (DRCS) (FR&CR) | both N.S. | both N.S. |  |  |  |  |  |  |
|  |  |  | Conflict (CBQ) (FR&CR) | both N.S. | both N.S. |  |  |  |  |  |  |
|  |  |  | Conflict (CBQ) (MR) | p=.000 | N.S. |  |  |  |  |  |  |
|  |  |  | Family behaviors (DFBC) (FR&MR&CR) - *supportive* | all N.S. | all N.S. |  |  |  |  |  |  |
|  |  |  | Family behaviors (DFBC) (FR&MR&CR) - *unsupportive* | all N.S. | all N.S. |  |  |  |  |  |  |
|  |  |  | Child adjustment (AIS) (FR&MR&CR) |  |  |  |  | all N.S. | all N.S. |  |  |
|  |  |  | Problem behavior (CBCL) (FR&CR) - *total* |  |  |  |  | both N.S. | both N.S. |  |  |
|  |  |  | Problem behavior (CBCL) (MR) - *total* |  |  |  |  | p=.024 | N.S. |  |  |
|  |  |  | Diabetes self-care (SCI) (FR&MR&CR) |  |  |  |  |  |  | all N.S. | all N.S. |
|  |  |  | Diabetes self-care (DMQ) (FR&MR&CR) |  |  |  |  |  |  | all N.S. | all N.S. |
|  |  |  | HbA1c (OM) |  |  |  |  |  |  | N.S. | N.S |
| Wysocki, 2006; Wysocki, 2007; Wysocki, 2008; | BFST-Diabetes (BFST-D),  educational control (EC), CAU | RM-ANOVA , group*time, adjusted  pre, post, 12 month FU;  (N=72) | Parent-child relationship (PARQ) (mean PR+CR) – *3 subscales* | EC/CAU: all N.S. |  |  |  |  |  |  |  |
|  |  |  | Diabetes conflict (DRCS) (mean PR+CR) | EC/CAU: both sign  (p NR) | EC/CAU: both N.S. |  |  |  |  |  |  |
|  |  |  | Negative reciprocity (IBC) (OR) | EC/CAU: both sign  (p NR) | EC: N.S.  CAU: sign (p NR) |  |  |  |  |  |  |
|  |  |  | Positive reciprocity (IBC) (OR) | EC/CAU: both sign  (p NR) | EC/CAU: both sign (p NR) |  |  |  |  |  |  |
|  |  |  | Problem-solving process (IBC) (OR) | EC/CAU: both N.S. | EC: N.S.  CAU: sign (p NR) |  |  |  |  |  |  |
|  |  |  | Problem resolution (IBC) (OR) | EC/CAU: both N.S. | |  |  |  |  |  |  |
|  |  |  | Negative communication (IBC) (OR) - *mother* | EC/CAU: both sign  (p NR) | EC/CAU: both sign (p NR) |  |  |  |  |  |  |
|  |  |  | Negative communication (IBC) (OR) - *father* | EC/CAU: both N.S. | EC/CAU: both N.S. |  |  |  |  |  |  |
|  |  |  | Positive communication (IBC) (OR) - *mother* | EC/CAU: both sign  (p NR) (I=, C-)^#^ | EC/CAU: sign (p NR) (I=, C-)^#^ |  |  |  |  |  |  |
|  |  |  | Positive communication (IBC) (OR) – *father&child* | EC/CAU: both N.S. | EC/CAU: both N.S. |  |  | EC/CAU: both N.S. | EC/CAU: both N.S. |  |  |
|  |  |  | Negative communication (IBC) (OR) - *child* |  |  |  |  | EC/CAU: both sign (p NR) | EC: N.S.  CAU: sign (p NR) |  |  |
|  |  |  | Diabetes self-care (DSMP) (I - mean PR+CR) |  |  |  |  |  |  | EC/CAU: both N.S. | EC/CAU: both N.S. |
|  |  |  | HbA1c (OM) |  |  |  |  |  |  | EC: N.S.  2006: CAU: N.S.  2007: CAU: p<.05 | EC: N.S.  CAU: p<.05 |
| Supplementary Table 1 – Extracted data of included studies on post and first follow up measures *(continued – 3)* | | | | | | | | | | | |
| Reports (first author, year) | Intervention, control | Reported effects (analyses, time-points, N) | Outcome (measure) (modality) - *(subscale)* | Parenting/  Family dynamics | | Parent | | Child | | Diabetes | |
|  |  |  |  | Post | FU | Post | FU | Post | FU | Post | FU |
| Harris 2015,  Riley 2015, Duke 2016 | BFST-D  (Skype/ face-to face collated),  no control | RM-ANOVA, time effects;  ^$^Results from subsample who filled out CDI (Riley 2015)  pre, post, 6 mo. FU  (N=90) | Miscarried helping (HHI) (PR) | p<.001, d=0.77 | p<.001, d=0.91 |  |  |  |  |  |  |
|  |  |  | Miscarried helping (HHI) (CR) | N.S. | p<.01, d=0.32 |  |  |  |  |  |  |
|  |  |  | Diabetes conflict (DFCS) (PR) | p<.05 ^$^ | N.S. ^$^ |  |  |  |  |  |  |
|  |  |  | Diabetes conflict (DFCS) (CR) | p<.001^$^ | p<.001^$^ |  |  |  |  |  |  |
|  |  |  | Conflict (CBQ) (PR) | p<.001, d=0.43 | p<.001, d=0.64 |  |  |  |  |  |  |
|  |  |  | Conflict (CBQ) (CR) | p<.01, d=0.35 | p<.01, d=0.52 |  |  |  |  |  |  |
|  |  |  | Parental adjustment (AIS) (PR) |  |  | p<.01, d=0.31 | p<.01, d=0.46 |  |  |  |  |
|  |  |  | Child adjustment (AIS) (CR) |  |  |  |  | N.S. | N.S. |  |  |
|  |  |  | Child depressive symptoms (CDI) (CR) |  |  |  |  | p=.01, d=0.39^$^ | p<.001, d=0.61^$^ |  |  |
|  |  |  | Diabetes self-care (DSMP) (I - PR) |  |  |  |  |  |  | p<.001, d=0.45 | p<.001, d=0.27 |
|  |  |  | Diabetes self-care (DSMP) (I - CR) |  |  |  |  |  |  | p<.01, d=0.44 | p<.001, d=0.39 |
|  |  |  | HbA1c (OM) |  |  |  |  |  |  | p<.01, d=0.40 | p<.01, d=0.37 |
| Lehmkuhl 2010 | Telehealth behavioral therapy (TBT) (phone),  waitlist control | Mixed model RM ANOVA, group*time  Pre, post;  (N=32)  Pilot | Family support (DFBS) (CR) - *warmth* | *p=.03* |  |  |  |  |  |  |  |
|  |  |  | Family support (DFBS) (CR) - *guidance* | N.S. |  |  |  |  |  |  |  |
|  |  |  | Unsupportive parenting (DFBC) (PR) | *p=.04* |  |  |  |  |  |  |  |
|  |  |  | Unsupportive parenting (DFBC) (CR) | N.S. |  |  |  |  |  |  |  |
|  |  |  | Parent T1D responsibilities (DFRQ) – *no responsibility* | N.S. |  |  |  |  |  |  |  |
|  |  |  | Diabetes self-care (DSMP) (I - PR&CR) |  |  |  |  |  |  | both N.S. |  |
|  |  |  | HbA1c (OM) |  |  |  |  |  |  | N.S. |  |
| Salcudean, 2024 | Systemic family psychotherapy (FT),  Individual therapy (IT),  CAU | Paired sample t-tests  Pre, post;  (N=64) | Parent-child relation (CPRS) (I – PR) - *closeness* | FT: p<.001, d=2.20  IT: NR  CAU: p<.001, d=4.42 |  |  |  |  |  |  |  |
|  |  |  | Parent-child relation (CPRS) (I – PR) - *conflict* | FT: p<.001, d=2.30  IT: NR  CAU: N.S. |  |  |  |  |  |  |  |
|  |  |  | HbA1c (PR) |  |  |  |  |  |  | FT: p<.001, d=2.20  IT: N.S.  CAU: p=.009, d=0.64 (increase) |  |

| Supplementary Table 1 – Extracted data of included studies on post and first follow up measures *(continued – 4)* | | | | | | | | | | | |
| --- | --- | --- | --- | --- | --- | --- | --- | --- | --- | --- | --- |
| Reports (first author, year) | Intervention, control | Reported effects (analyses, time-points, N) | Outcome (measure) (modality) - *(subscale)* | Parenting/  Family dynamics | | Parent | | Child | | Diabetes | |
|  |  |  |  | Post | FU | Post | FU | Post | FU | Post | FU |
| **Multisystemic therapy (MST)** | | | | | | | | | | | |
| Ellis 2004 | MST,  CAU | Paired t-tests per group  pre, post;  (N=25)  Pilot | Diabetes self-care (DMS) (PR&CR) |  |  |  |  |  |  | I: both N.S.;  C: both N.S. |  |
|  |  |  | BGM frequency (OM) |  |  |  |  |  |  | I: N.S.; C: N.S. |  |
|  |  |  | Diabetes self-care (24 hour recall) (I - CR) – *insulin/eating* |  |  |  |  |  |  | I: both N.S.;  C: both N.S. |  |
|  |  |  | Diabetes self-care (24 hour recall) (I - CR) – *BGM* |  |  |  |  |  |  | I: p<.05; C: N.S. |  |
|  |  |  | Emergency room visits (OM) |  |  |  |  |  |  | I: N.S.; C: N.S. |  |
|  |  |  | Hospital admissions (OM) |  |  |  |  |  |  | I: p<.05;  C: N.S. |  |
|  |  |  | GHb (OM) |  |  |  |  |  |  | I: p<.01, d=0.91;  C: N.S. |  |
| Ellis 2005a; Ellis 2005b; Ellis 2007a; Ellis 2007b;  Naar-King 2007 | MST,  CAU | ^$^Mixed design ANOVA (group*time);  ^$$^General linear mixed model (group*time);  ^$$$^ANOVA on change scores  Pre, post, 12 mo. FU  (N=127) | Caregiver support (DFBC) (mean PR+CR) - *supportive* | N.S.^$$$^ |  |  |  |  |  |  |  |
|  |  |  | Family relationships (FES) (mean PR+CR) – *Family Relationship Index* | N.S. ^$$$^ |  |  |  |  |  |  |  |
|  |  |  | Parental overestimation of child responsibilities (DFRQ, adapted) (PR+CR combined) | p<.05, d=0.46^$$^ | p<.05, d=0.44^$$^ |  |  |  |  |  |  |
|  |  |  | Diabetes distress (DSQ) (CR) |  |  |  |  | p=.004^$^ |  |  |  |
|  |  |  | Diabetes self-care (24 hour recall) (I - CR) – *insulin/eating* |  |  |  |  |  |  | both N.S. ^$^ |  |
|  |  |  | Diabetes self-care (24 hour recall) (I - CR)– *BGM* |  |  |  |  |  |  | p=.011, d=0.83^$^ (I+, C=) ^#^ |  |
|  |  |  | Diabetes self-care – daily BGM frequency (OM) |  |  |  |  |  |  | p=.001, d=1.09^$^ (I+,C=)^#^;  p<.01^$$^,  (I+, C-)^#^;  p<.001^$$$^, (I+, C-)^#^ | N.S.^$$^ |
|  |  |  | Emergency room visits (OM) |  |  |  |  |  |  | N.S. ^$^ |  |
|  |  |  | Hospital admission (OM) |  |  |  |  |  |  | p=.014, d=0.63^$^  (I-, C+)^#^ |  |
|  |  |  | DKA admission rate (relative DKA drop in MST /relative DKA drop in control) (OM) |  |  |  |  |  |  | p<.001, relative rate ratio 0.26 | p=.004, relative rate ratio 0.39^$$^ |
|  |  |  | HbA1c (OM) |  |  |  |  |  |  | N.S. ^$^;  p<.05, d=0.41^$$^  (I-, C=)^#^;  N.S.^$$$^ | N.S.^$$^ |
| Supplementary Table 1 – Extracted data of included studies on post and first follow up measures *(continued – 5)* | | | | | | | | | | | |
| Reports (first author, year) | Intervention, control | Reported effects (analyses, time-points, N) | Outcome (measure) (modality) - *(subscale)* | Parenting/  Family dynamics | | Parent | | Child | | Diabetes | |
|  |  |  |  | Post | FU | Post | FU | Post | FU | Post | FU |
| Ellis 2012 | MST,  telephone support | Linear regression, group effect  Pre, post, 12 mo. FU;  (N=146) | Diabetes self-care (DMS) (PR) |  |  |  |  |  |  | p<.05 | p<.05 |
|  |  |  | Diabetes self-care (DMS) (CR) |  |  |  |  |  |  | N.S. | N.S. |
|  |  |  | HbA1c (OM) |  |  |  |  |  |  | p<.05 | N.S. |
|  |  |  | Adverse diabetes events (measure NR) |  |  |  |  |  |  | N.S. | N.S. |
| Ellis 2019 | RFC,  CAU | Paired t-tests per group  Pre, post  (N=50)  Pilot | Quality of life (DQOL) (CR) |  |  |  |  | I: p=.001;  C: N.S. |  |  |  |
|  |  |  | Diabetes self-care (DMS) (PR&CR) |  |  |  |  |  |  | I: both N.S.;  C: both N.S. |  |
|  |  |  | Diabetes self-care – BGM frequency (OM) |  |  |  |  |  |  | I: N.S.; C: N.S. |  |
|  |  |  | HbA1c (OM) |  |  |  |  |  |  | I: p=.05; C: N.S. |  |
| **Routine care integrated interventions** | | | | | | | | | | | |
| Anderson 1999 | Office teamwork (TW),  attention control/ CAU (combined) | ^$^RM-ANOVA group*time;  ^$$^Chi^2^  ^$$$^t-test on FU measure  pre, post; 12 mo. FU  (N=82) | Unsupportive parenting (DFBC) (PR) | p<.02^$^ |  |  |  |  |  |  |  |
|  |  |  | Deterioration in involvement (self-developed) (I – PR+CR)– *insulin* | p<.03^$$^ |  |  |  |  |  |  |  |
|  |  |  | Deterioration in involvement (self-developed) (I – PR+CR)– *monitoring* | N.S. ^$$^ |  |  |  |  |  |  |  |
|  |  |  | Diabetes conflict (DFCS) (PR) | p<.02^$^ |  |  |  |  |  |  |  |
|  |  |  | HbA1c (OM) |  |  |  |  |  |  |  | N.S.^$$$^ |
| Laffel 2003 | Teamwork intervention,  CAU | ^$^ paired t-tests per group;  ^$$^ t-test on post measure  Pre-post;  (N=100) | Parent T1D responsibilities (DFRQ) (PR&CR) | I: both N.S. ^$^  C: both N.S.^$^ |  |  |  |  |  |  |  |
|  |  |  | Parent T1D responsibilities (self-developed) (I - PR+CR combined) – *BGM and insulin injection* | I: NR  C: NR |  |  |  |  |  |  |  |
|  |  |  | Diabetes conflict (DFCS) (PR&CR) | I: both N.S. ^$^  C: both N.S.^$^ |  |  |  |  |  |  |  |
|  |  |  | Quality of life (PedsQL) (PR&CR) |  |  |  |  | I: both N.S. ^$^  C: both N.S.^$^ |  |  |  |
|  |  |  | Diabetes self-care (rating scale) (CLR) |  |  |  |  |  |  | NR |  |
|  |  |  | BGM frequency (I - PR+CR) |  |  |  |  |  |  | N.S. ^$^ |  |
|  |  |  | HbA1c^$^ (OM) |  |  |  |  |  |  | p<.05^$$^ (I=, C-)^#^ |  |

| Supplementary Table 1 – Extracted data of included studies on post and first follow up measures *(continued – 6)* | | | | | | | | | | | |
| --- | --- | --- | --- | --- | --- | --- | --- | --- | --- | --- | --- |
| Reports (first author, year) | Intervention, control | Reported effects (analyses, time-points, N) | Outcome (measure) (modality) - *(subscale)* | Parenting/  Family dynamics | | Parent | | Child | | Diabetes | |
|  |  |  |  | Post | FU | Post | FU | Post | FU | Post | FU |
| Svoren 2003 | Family psychoeducation (PE) + care ambassador (CA),  CA only/ CAU (combined) | t-tests on post measure, I vs. CA/CAU combined  Pre, post (at 24 mo.);  (N=191) | Severe hypoglycemia’s (multiple measures) (I -chart review-BGM/HIS) - *decrease* |  |  |  |  |  |  | p=.02, 25% less vs CA/CAU |  |
|  |  |  | Hospital admissions (BGM/HIS) (I -chart review-BGM/HIS) - *decrease* |  |  |  |  |  |  | p=.04, 40% less vs CA/CAU |  |
|  |  |  | Emergency department visits (I -chart review-BGM/HIS) - *decrease* |  |  |  |  |  |  | p=.004, 40% less vs CA/CAU |  |
|  |  |  | HbA1c – mean past 24 months (OM) |  |  |  |  |  |  | N.S. |  |
| Katz 2014 | Family psychoeducation (PE) + care ambassador (CA),  CA only, CAU | MANOVA, adjusted  Pre, post;  (N=101) | Parent T1D responsibilities (DFRQ) (mean PR+CR) | CA: p=.04; CAU: N.S. |  |  |  |  |  |  |  |
|  |  |  | Diabetes conflict (DFCS) (PR&CR) | CA/CAU: all N.S. |  |  |  |  |  |  |  |
|  |  |  | Quality of life (PedsQL-Generic) (PR&CR) |  |  |  |  | CA/CAU: both N.S. |  |  |  |
|  |  |  | HbA1c (OM) |  |  |  |  |  |  | CA/CAU: both N.S. |  |
|  |  |  | BGM (CLR or PR or CR) |  |  |  |  |  |  | CA/CAU: both N.S. |  |
| Holmes, 2014 | Family teamwork coping skills training  (Family TW CST),  educational control | Growth curve analyses, group*year rate of change over time, adjusted  Pre, post, FU every 3.5 mo. up to 1.5 years post termination  (analyses also include later time-points)  (N=226) | Monitoring (PMDC) (PR) | N.S. | |  |  |  |  |  |  |
|  |  |  | Effectiveness monitoring beliefs (PIS) (PR)- *outcome expectations* | N.S. | |  |  |  |  |  |  |
|  |  |  | Diabetes conflict (DFCS) (mean PR+CR) | N.S. | |  |  |  |  |  |  |
|  |  |  | Conflict general (FES) (mean PR+CR) | N.S. | |  |  |  |  |  |  |
|  |  |  | Self-efficacy (SEDSM) (CR) |  |  |  |  | N.S. | |  |  |
|  |  |  | Quality of life (PedsQL- Diabetes) (mean PR+CR) |  |  |  |  | N.S. | |  |  |
|  |  |  | Diabetes self-care (DBRS) (mean PR+CR) |  |  |  |  |  |  | *p=.011, B= -0.02* | |
|  |  |  | HbA1c (OM) |  |  |  |  |  |  | *p=.05, B=0.17* | |
| Murphy 2007 | FACTS,  waitlist control | t-tests on change scores  Pre, post (at 12 mo.);  (N=78) | Parent T1D responsibilities (DFRQ) (PR+CR combined) | NR |  |  |  |  |  |  |  |
|  |  |  | Quality of life (PedsQL) (PR&CR) |  |  |  |  | both N.S. |  |  |  |
|  |  |  | Diabetes distress (PAID) (CR or PR) |  |  |  |  | N.S. |  |  |  |
|  |  |  | HbA1c (OM) |  |  |  |  |  |  | N.S. |  |

| Supplementary Table 1 – Extracted data of included studies on post and first follow up measures *(continued – 7)* | | | | | | | | | | | |
| --- | --- | --- | --- | --- | --- | --- | --- | --- | --- | --- | --- |
| Reports (first author, year) | Intervention, control | Reported effects (analyses, time-points, N) | Outcome (measure) (modality) - *(subscale)* | Parenting/  Family dynamics | | Parent | | Child | | Diabetes | |
|  |  |  |  | Post | FU | Post | FU | Post | FU | Post | FU |
| Murphy 2012 | FACTS,  CAU | ^$^RM-ANOVA (effect NR), adjusted;  ^$$^Wilcoxon’s test  ^*^Pre, 12 mo. ^**^FU, 18 mo. FU^;^  (N=305) | Parent T1D responsibilities (DFRQ) (respondent NR) |  | N.S.^$*^ |  |  |  |  |  |  |
|  |  |  | Diabetes distress (PAID) (PR) |  |  |  | N.S.^$*^ |  |  |  |  |
|  |  |  | Quality of life (DQOLY) (respondent NR)– *3 subscales* |  |  |  |  |  | N.S.^$*^ |  |  |
|  |  |  | Wellbeing (WHO HBSC) (respondent NR) |  |  |  |  |  | N.S.^$*^ |  |  |
|  |  |  | Severe hypoglycemia’s (OM) |  |  |  |  |  |  |  | N.S.^$**^ |
|  |  |  | Hospital admissions (OM) |  |  |  |  |  |  |  | N.S.^$**^ |
|  |  |  | % adjusting insulin frequently/always (single item) (respondent NR)– *based on snacks/meals/BG results* |  |  |  |  |  |  |  | I: all p=.012^$$**^,  C: all N.S. ^$$**^ |
|  |  |  | HbA1c (OM) |  |  |  |  |  |  |  | N.S.^$**^ |
| Nansel, 2009 | WE-CAN,  CAU | Analyses NR  Pre, post;  (N=132)  Feasibility | Parent child conflict (DFCS) (PR&CR) | both N.S. |  |  |  |  |  |  |  |
|  |  |  | Parent T1D responsibilities (DFRQ) (PR&CR) | both N.S. |  |  |  |  |  |  |  |
|  |  |  | Quality of life (PedsQL- Generic) (PR&CR) |  |  |  |  | both N.S. |  |  |  |
|  |  |  | Quality of life (PedsQL- Diabetes) (PR&CR) |  |  |  |  | both N.S. |  |  |  |
|  |  |  | Diabetes self-care (DSMP) (I - PR&CR) |  |  |  |  |  |  | both N.S. |  |
|  |  |  | BGM frequency (OM) |  |  |  |  |  |  | N.S. |  |
|  |  |  | HbA1c (OM) |  |  |  |  |  |  | N.S. |  |
| Nansel 2012, Nansel 2015,  Gee 2017,  Temmen, 2022  Lu, 2023 | WE-CAN,  CAU | ^$^t-test on change;  ^$$^ANOVA;  ^$$$^ANCOVA, adjusted  Pre, post;  (N=390) | Parent T1D responsibilities (DFRQ) (PR&CR) | both N.S. ^$$$^ |  |  |  |  |  |  |  |
|  |  |  | Collaborative involvement (CPI) (CR) | N.S. ^$$$^ |  |  |  |  |  |  |  |
|  |  |  | Diabetes conflict (DFCS) (PR&CR) | both N.S. ^$$$^ |  |  |  |  |  |  |  |
|  |  |  | Diabetes self-care (DSMP) (I - PR&CR) |  |  |  |  |  |  | both N.S. ^$^ |  |
|  |  |  | BGM frequency (OM) |  |  |  |  |  |  | *p=.03^$^* |  |
|  |  |  | Hypoglycemia(treated orally/parenteral) (PR or CR) |  |  |  |  |  |  | N.S. ^$$^ |  |
|  |  |  | HbA1c (OM) |  |  |  |  |  |  | p=.03^$^ |  |
| Monaghan 2015 | Checking in,  no control | Paired t-tests  Pre, 12 week FU;  (N=30)  Pilot | Monitoring (PMDC) (PR) |  | N.S. |  |  |  |  |  |  |
|  |  |  | Collaborative involvement (CPI) (CR) |  | N.S. |  |  |  |  |  |  |
|  |  |  | Diabetes conflict (DFCS) (PR&CR) |  | both N.S. |  |  |  |  |  |  |
|  |  |  | Diabetes self-care (SCI) (PR&CR) |  |  |  |  |  |  |  | both N.S. |
|  |  |  | HbA1c (OM) |  |  |  |  |  |  |  | N.S. |
|  |  |  | BGM frequency (OM) |  |  |  |  |  |  |  | N.S. |
|  |  |  | Mean BG level (prior 30 days) (OM) |  |  |  |  |  |  |  | N.S. |

| Supplementary Table 1 – Extracted data of included studies on post and first follow up measures *(continued – 8)* | | | | | | | | | | | |
| --- | --- | --- | --- | --- | --- | --- | --- | --- | --- | --- | --- |
| Reports (first author, year) | Intervention, control | Reported effects (analyses, time-points, N) | Outcome (measure) (modality) - *(subscale)* | Parenting/  Family dynamics | | Parent | | Child | | Diabetes | |
|  |  |  |  | Post | FU | Post | FU | Post | FU | Post | FU |
| Ellis, 2017 | 3MS,  attention control | Paired t-tests per group  pre, post;  (N=43)  Feasibility | Monitoring (PMDC-R) (PR) – *direct observation* | I: N.S.; C: N.S. |  |  |  |  |  |  |  |
|  |  |  | Conflict (PARQ) (PR) – *global distress* | I: N.S.; C: N.S. |  |  |  |  |  |  |  |
|  |  |  | Knowledge need of monitoring (self-developed) (PR) |  |  | I: p=.01, d=1.48  C: p=.01, d=0.55 |  |  |  |  |  |
|  |  |  | Motivation for monitoring (RRR) (PR) |  |  | I: N.S.; C: N.S. |  |  |  |  |  |
|  |  |  | Self-efficacy for monitoring (RRR) (PR) |  |  | I: N.S.; C: N.S. |  |  |  |  |  |
|  |  |  | HbA1c (OM) |  |  |  |  |  |  | I: p=.05, d=-0.42;  C: N.S. |  |
| Ellis 2024, Knauft 2024 | 3MS,  CAU | RM linear mixed effects (LME) regression,  pre, post, 6 mo. FU | HbA1c (OM) |  |  |  |  |  |  | N.S. | N.S. |
| **Combined parent child interventions** | | | | | | | | | | | |
| Ambrosino 2008,  Grey 2009,  Grey 2011 | Coping skills training (CST),  group education | 2008: Mixed model group*time;  pre, 1 & 3 mo. FU (N=111)  2009: Regression, group*time, adjusted;  Pre, 3, 6 mo. FU;  (N=111)  2011: Regression, group*time;  pre, 3, mo. FU;  (N=181) | Parent T1D responsibilities (DRCS) (PR) – *2 subscales* | 2008/2011: all N.S. | |  |  |  |  |  |  |
|  |  |  | Family functioning (FACES II) (PR) - *adaptability* | 2008: p=.01, d=.28 or N.S. (inconsistent in report) | |  |  |  |  |  |  |
|  |  |  | Family functioning (FACES II) (PR) - *cohesion* | 2008: N.S. | |  |  |  |  |  |  |
|  |  |  | Family support (DFBS) (CR) – *2 subscales* | 2008/2009: all N.S. | |  | |  |  |  |  |
|  |  |  | Coping  (ICIDDM- Parent) (PR) – *2 subscales* |  |  | 2008: all N.S.; 2011: mean N.S. | |  |  |  |  |
|  |  |  | Depressive symptoms (CES-D) (PR) |  |  | 2008: N.S. | |  |  |  |  |
|  |  |  | Quality of Life (PDQOL) (PR) - *impact* |  |  | 2011: N.S. | |  | |  |  |
|  |  |  | Coping (ICIDDM - Child) (CR) – *2 subscales* |  |  |  |  | 2008/2009: all N.S. | |  |  |
|  |  |  | Self-efficacy (SEDS) (CR) – *diabetes* |  |  |  |  | 2008/2009: both N.S. | |  |  |
|  |  |  | Self-efficacy (SEDS) (CR) – *2 subscales* |  |  |  |  | 2008: all N.S. | |  |  |
|  |  |  | Depressive symptoms (CDI) (CR) |  |  |  |  | 2009: N.S. | |  |  |
|  |  |  | Quality of life (DQOL) (CR) – *3 subscales* |  |  |  |  | 2008/2009: all N.S. | |  |  |
|  |  |  | HbA1c (OM) |  |  |  |  |  |  | 2008/2009/2011: N.S. | |

| Supplementary Table 1 – Extracted data of included studies on post and first follow up measures *(continued – 9)* | | | | | | | | | | | |
| --- | --- | --- | --- | --- | --- | --- | --- | --- | --- | --- | --- |
| Reports (first author, year) | Intervention, control | Reported effects (analyses, time-points, N) | Outcome (measure) (modality) - *(subscale)* | Parenting/  Family dynamics | | Parent | | Child | | Diabetes | |
|  |  |  |  | Post | FU | Post | FU | Post | FU | Post | FU |
| Opipari 2005 *(conference abstract)* | Kicking in diabetes support (K.I.D.S.),  no control | RM ANOVA  Pre, post, 12 mo. FU  (N=29) | Conflict (respondent NR) |  | p<.05 |  |  |  |  |  |  |
|  |  |  | Diabetes knowledge (respondent NR) |  |  |  |  |  | p<.01 |  |  |
|  |  |  | Adolescent responsibility (respondent NR) |  |  |  |  |  | p<.01 |  |  |
|  |  |  | HbA1c (NR |  |  |  |  |  |  |  | N.S. |
| Kichler 2013 | Kicking in diabetes support (K.I.D.S.),  waitlist control | MANCOVA group effect, adjusted  Pre, post, 4 mo. FU;  (N=30)  Pilot | Parent T1D responsibilities (DRCS) (PR&CR) | both N.S. |  |  |  |  |  |  |  |
|  |  |  | Family impact (PedsQL- Family Impact) (PR) |  |  | NR |  |  |  |  |  |
|  |  |  | Readiness to change (RCBRS) (PR) |  |  | N.S. |  |  |  |  |  |
|  |  |  | Psychological distress (BSI-18) (PR) |  |  | N.S. |  |  |  |  |  |
|  |  |  | Readiness to change (RCBRS) (CR) |  |  |  |  | N.S. |  |  |  |
|  |  |  | Quality of life (PedsQL Diabetes) (PR) |  |  |  |  | N.S. |  |  |  |
|  |  |  | Quality of life (PedsQL Diabetes) (CR) |  |  |  |  | *p<.05, ES=0.34* |  |  |  |
|  |  |  | Quality of life (PedsQL Generic) (PR) – *psychosocial health* |  |  |  |  | N.S. |  |  |  |
|  |  |  | Quality of life (PedsQL Generic) (CR) – *psychosocial health* |  |  |  |  | *p<.05, ES=0.31* |  |  |  |
|  |  |  | Child behavior (BASC-2) (PR) – *behavioral & emotional symptoms index* |  |  |  |  | all N.S. |  |  |  |
|  |  |  | Diabetes self- care (SCI) (PR) |  |  |  |  |  |  | N.S. |  |
|  |  |  | Diabetes self-care (SCI) (CR) |  |  |  |  |  |  | NR |  |
|  |  |  | Health care utilization (OM) |  |  |  |  |  |  | N.S. |  |
|  |  |  | HbA1c (OM) |  |  |  |  |  |  | N.S. |  |
| Kichler 2021 | Kicking in diabetes support (K.I.D.S.),  no control | Paired t-tests  Pre, post, 6 mo. FU;  (N=38) | Parent T1D responsibilities (DFRQ) (PR) | p=.016, d=0.46 | N.S. |  |  |  |  |  |  |
|  |  |  | Readiness to change responsibility (RCBRS) (PR) |  |  | p=.005, d=0.45 | p=.005, d=0.53 |  |  |  |  |
|  |  |  | Diabetes self-care (SCI) (PR) |  |  |  |  |  |  | p=.017, d=0.38 | p=.006, d=0.50 |
|  |  |  | No. of T1D clinic visits (OM) |  |  |  |  |  |  | p=.028, d=0.37 | N.S. |
|  |  |  | No. of T1D ER visits (OM) |  |  |  |  |  |  | p=.007, d=NR | N.S. |
|  |  |  | No. of T1D related hospitalizations (OM) |  |  |  |  |  |  | N.S. | N.S. |
|  |  |  | HbA1c (OM) |  |  |  |  |  |  | p=.021, d=0.54 | p=.020, d=0.41 |

| Supplementary Table 1 – Extracted data of included studies on post and first follow up measures *(continued – 10)* | | | | | | | | | | | |
| --- | --- | --- | --- | --- | --- | --- | --- | --- | --- | --- | --- |
| Reports (first author, year) | Intervention, control | Reported effects (analyses, time-points, N) | Outcome (measure) (modality) - *(subscale)* | Parenting/  Family dynamics | | Parent | | Child | | Diabetes | |
|  |  |  |  | Post | FU | Post | FU | Post | FU | Post | FU |
| Satin 1989 | MF and MF+S,  CAU | ^$^t-test on change score;  ^$$^ANOVA group*time (Bonferroni adjusted p-values)  pre, post, 6 mo. FU (HbA1c only)  (N=32) | Family functioning (FES) (PR) - *10 subscales* | MF+S: all N.S.^$$^;  MF: all N.S. ^$$^ |  |  |  |  |  |  |  |
|  |  |  | Family functioning (FES) (CR) - *10 subscales* | MF+S: all N.S.^$$^;  MF: all N.S. ^$$^ |  |  |  |  |  |  |  |
|  |  |  | Attitude CwD (Semantic differentials) (FR&MR&CR) |  |  | MF+S: both N.S.^$^  MF: both N.S. ^$^ |  | MF+S: p<.01^$^ (I=, C-)^#^;  MF: p<.05^$^ |  |  |  |
|  |  |  | Attitude parent of CwD (Semantic differentials) (FR&MR&CR) |  |  | MF+S: both N.S.^$$^  MF: both N.S. ^$$^ |  | N.S. ^$$^ |  |  |  |
|  |  |  | Attitude family of CwD (Semantic differentials) (FR&MR&CR) |  |  | MF+S: both N.S.^$$^  MF: both N.S. ^$$^ |  | N.S. ^$$^ |  |  |  |
|  |  |  | Diabetes self-care (1-item) (FR&MR) |  |  |  |  |  |  | MF+S: both N.S. ^$^  MF: both N.S. ^$^ |  |
|  |  |  | HbA1c (OM) |  |  |  |  |  |  | MF+S: p<.05^$^  (I-; C+)^#^;  MF: N.S. ^$^ | MF+S: N.S. ^$^  MF: N.S. ^$^ |
| Carpenter 2014 | Multifamily group problem solving intervention (MF group PS),  no control | ANCOVA, time effect, adjusted  pre, post  (N=67) | HbA1c (OM) |  |  |  |  |  |  | N.S. |  |
| Patel 2022 *(conference abstract)* | Counseling,  no control | Analyses NR  Pre, post  (N=NR) | Quality of life (respondent NR) |  |  |  |  | NR |  |  |  |
|  |  |  | Psychological stress (respondent NR) |  |  |  |  | NR |  |  |  |
|  |  |  | Social wellness (respondent NR) |  |  |  |  | NR |  |  |  |
|  |  |  | HbA1c respondent (NR) |  |  |  |  |  |  | NR |  |
| **Stand-alone parent trainings** | | | | | | | | | | | |
| Doherty 2013 | Triple P,  CAU | ANCOVA, adjusted  pre, post  (N=79) | Ineffective discipline strategies (PS) (PR) | p=.039 |  |  |  |  |  |  |  |
|  |  |  | Diabetes conflict (DFCS) (PR) | p=.008 |  |  |  |  |  |  |  |
|  |  |  | Illness related stress (PIP) (PR) |  |  | N.S. |  |  |  |  |  |
|  |  |  | Parenting confidence (PSOC) (PR) |  |  | p=.006 |  |  |  |  |  |
|  |  |  | Problematic child behavior (ECBI) (PR) - *intensity* |  |  |  |  | p=.008 |  |  |  |

| Supplementary Table 1 – Extracted data of included studies on post and first follow up measures *(continued – 11)* | | | | | | | | | | | |
| --- | --- | --- | --- | --- | --- | --- | --- | --- | --- | --- | --- |
| Reports (first author, year) | Intervention, control | Reported effects (analyses, time-points, N) | Outcome (measure) (modality) - *(subscale)* | Parenting/  Family dynamics | | Parent | | Child | | Diabetes | |
|  |  |  |  | Post | FU | Post | FU | Post | FU | Post | FU |
| Westrupp 2014 | Triple P,  CAU | Linear regression  pre, post, 12 mo. FU  (N=76) | Ineffective discipline strategies (PS) (PR) - *total* | N.S. | N.S. |  |  |  |  |  |  |
|  |  |  | Conflict over parenting (PPC) (PR) | p=.047, ES=0.44 | N.S. |  |  |  |  |  |  |
|  |  |  | Diabetes conflict (DFCS) (PR) | N.S. | N.S. |  |  |  |  |  |  |
|  |  |  | Marital quality (RQI) (PR) | N.S. | N.S. |  |  |  |  |  |  |
|  |  |  | Child behavior (BASC-2) (PR) – *parent rating scale –* *internalize & externalize* |  |  |  |  | both N.S. | both N.S. |  |  |
|  |  |  | Disruptive behavior (ECBI) (PR) – *intensity & number* |  |  |  |  | both N.S. | both N.S. |  |  |
|  |  |  | Depressive symptoms (DASS) (PR) |  |  | p=.007, ES=0.44 | N.S. |  |  |  |  |
|  |  |  | Anxiety symptoms (DASS) (PR) |  |  | p=.034, ES=0.44 | N.S. |  |  |  |  |
|  |  |  | Stress symptoms (DASS) (PR) |  |  | p=.017, ES=0.51 | N.S. |  |  |  |  |
|  |  |  | View parenting role (PSOC) (PR) - *satisfaction* |  |  | N.S. | N.S. |  |  |  |  |
|  |  |  | View parenting role (PSOC) (PR) – *self-efficacy* |  |  | N.S. | N.S. |  |  |  |  |
|  |  |  | HbA1c (OM) |  |  |  |  |  |  | N.S. | N.S. |
| Arkan 2020 | Triple P,  no control | Paired t-tests  pre, post  (N=32) | Parenting (PARI) (PR) - *overprotection* | p=.000 |  |  |  |  |  |  |  |
|  |  |  | Parenting (PARI) (PR) – *democratic approach* | p=.001 |  |  |  |  |  |  |  |
|  |  |  | Parenting (PARI) (PR) – *denial of housewife role* | N.S. |  |  |  |  |  |  |  |
|  |  |  | Parenting (PARI) (PR) – *marriage conflict* | p=.000 |  |  |  |  |  |  |  |
|  |  |  | Parenting (PARI) (PR) – *strict discipline* | p=.000 |  |  |  |  |  |  |  |
|  |  |  | Conflict (CBAQ) (PR&CR) | both p=.000 |  |  |  |  |  |  |  |
|  |  |  | Parent mental problems (GHQ-12) (PR) |  |  | p=.000 |  |  |  |  |  |
|  |  |  | Child mental problems (SDQ) (CR) |  |  |  |  | p=.000 |  |  |  |
|  |  |  | HbA1c (OM) |  |  |  |  |  |  | N.S. |  |
| May 2017 | Individualized feedback,  educational control | RM-ANOVA group*time  ^$^ corrected for broad positive communication  Pre, post  (N=79)  Pilot | Perceived closeness of parent (IOS) (CR) | N.S. |  |  |  |  |  |  |  |
|  |  |  | Parental positive communication (self-developed) - *total* (OR) | N.S. |  |  |  |  |  |  |  |
|  |  |  | Specific person centered communication (self-developed) (OR) | p=.02, eta_p_^2^=.05^$^ |  |  |  |  |  |  |  |
|  |  |  | Parental critical communication (self-developed) (OR) | N.S. |  |  |  |  |  |  |  |
|  |  |  | Parental intimacy during conversation (MIE) (CR) | N.S. |  |  |  |  |  |  |  |
|  |  |  | Child intimacy during conversation (MIE) (PR) |  |  |  |  | N.S. |  |  |  |
|  |  |  | Perceived closeness of child (IOS) (PR) |  |  |  |  | N.S. |  |  |  |

| Supplementary Table 1 – Extracted data of included studies on post and first follow up measures *(continued –12)* | | | | | | | | | | | |
| --- | --- | --- | --- | --- | --- | --- | --- | --- | --- | --- | --- |
| Reports (first author, year) | Intervention, control | Reported effects (analyses, time-points, N) | Outcome (measure) (modality) - *(subscale)* | Parenting/  Family dynamics | | Parent | | Child | | Diabetes | |
|  |  |  |  | Post | FU | Post | FU | Post | FU | Post | FU |
| Sassmann 2012 | DELFIN, waitlist  control | Linear model group*time^$^;  pre, post, 12 mo. FU  (N=37)  Feasibility | Ineffective discipline strategies (PS) (PR) – total | N.S. | NR |  |  |  |  |  |  |
|  |  |  | Positive parenting (QEB) (PR) | N.S. | NR |  |  |  |  |  |  |
|  |  |  | Psychological symptoms (DASS) (PR) – *total* |  |  | N.S. | NR |  |  |  |  |
|  |  |  | Behavioral difficulties (SDQ) (PR) – *total difficulties* |  |  |  |  | N.S. | NR |  |  |
|  |  |  | Prosocial behavior (SDQ) (PR) – *prosocial behavior* |  |  |  |  | N.S. | NR |  |  |
|  |  |  | HbA1c (OM) |  |  |  |  |  |  | p=.029 (I=, C+) | NR |
| Mitchell, 2022 | Healthy Living Triple P,  CAU | Mixed- model RM group* time;  Pre, post, 6 mo. FU;  (N=50) | Parenting (APQ) (PR) – *3 subscales* | all N.S. | |  |  |  |  |  |  |
|  |  |  | Parenting (APQ) (PR) - *corporal punishment* | p=.049, b=-0.33 (I+, C=) | |  |  |  |  |  |  |
|  |  |  | Parenting (APQ) (CR) – *4 subscales* | all N.S. | |  |  |  |  |  |  |
|  |  |  | Positive parenting (MOS) (OR) | N.S. | |  |  |  |  |  |  |
|  |  |  | Aversive parenting (MOS) (OR) | N.S. | |  |  |  |  |  |  |
|  |  |  | Confidence dealing with difficulties (CAPES) (PR) - *confidence* |  | | N.S. | |  |  |  |  |
|  |  |  | Self-efficacy (SEDS) (PR) |  | | N.S. | |  |  |  |  |
|  |  |  | Self-efficacy (DBC) (PR) - *confidence* |  | | N.S. | |  |  |  |  |
|  |  |  | Parenting stress (PSI-SF) (PR) |  | | N.S. | |  |  |  |  |
|  |  |  | Adjustment (PECIS) (PR) – *4 subscales* |  | | all N.S. | |  |  |  |  |
|  |  |  | Family impact (PedsQL Family Impact) (PR) |  | | N.S. | |  |  |  |  |
|  |  |  | Behavioral difficulties (CAPES) (PR) - *Intensity* |  | |  |  | N.S. | |  |  |
|  |  |  | Quality of life (PedsQL Generic) (PR) |  | |  |  | N.S. | |  |  |
|  |  |  | Child behavior (DBC) (PR) - *extent* |  | |  |  | N.S. | |  |  |
|  |  |  | Positive behavior (MOS) (OR) |  | |  |  | N.S. | |  |  |
|  |  |  | HbA1c (OM) |  | |  |  |  |  | N.S. | |
| Jones 2023 | PRIORITY,  waitlist control | Effect sizes and CI, no exact p-values;  pre, post, 3 mo. FU  (N=89);  Feasibility | Wellbeing (WEMWBS) (PR) |  |  | N.S. | N.S. |  |  |  |  |
|  |  |  | Diabetes distress (PAID) (PR) |  |  | N.S. | N.S. |  |  |  |  |
|  |  |  | Disordered eating (DEPS-R) (PR&CR) |  |  |  |  | both N.S. | both N.S. |  |  |
|  |  |  | Child eating (CEBQ) (PR) – *7 subscales* |  |  |  |  | all N.S. | all N.S. |  |  |
|  |  |  | Child eating (CEBQ) (PR) – *satiety responsiveness* |  |  |  |  | N.S. | d=0.55; p<.05 |  |  |

| Supplementary Table 1 – Extracted data of included studies on post and first follow up measures *(continued – 13)* | | | | | | | | | | | |
| --- | --- | --- | --- | --- | --- | --- | --- | --- | --- | --- | --- |
| Reports (first author, year) | Intervention, control | Reported effects (analyses, time-points, N) | Outcome (measure) (modality) - *(subscale)* | Parenting/  Family dynamics | | Parent | | Child | | Diabetes | |
|  |  |  |  | Post | FU | Post | FU | Post | FU | Post | FU |
| SENCE study group, 2021; Commissariat, 2023;  Van Name, 2023 | Family Behavioral Intervention (FBI) + CGM,  CGM only | Linear (mixed effect) regression, adjusted; Fisher’s exact test  Linear regression, adjusted  Pre, post, 12 mo. FU  p-values adjusted  (N=87) | Diabetes burden (PAID) (PR) |  |  | p=.008 | N.S. |  |  |  |  |
|  |  |  | Fear of hypoglycemia (HFS-PYC) (PR) |  |  | p=.037 | N.S. |  |  |  |  |
|  |  |  | Diabetes Family Impact Scale (DFI-S) (PR) |  |  | N.S. | N.S. |  |  |  |  |
|  |  |  | Parent wellbeing (WHO-5) (PR) |  |  | N.S. | N.S. |  |  |  |  |
|  |  |  | TIR/hyperglycemia/hypoglycemia (OM) |  |  |  |  |  |  | all N.S. | all N.S. |
|  |  |  | HbA1c (OM) |  |  |  |  |  |  | N.S. | N.S. |
| Rothman Kabir, 2022 | New Authority (NA) training,  no control | Mixed models (effects split out by informant if sign. family member * time interaction)  Pre, post, 5 mo. FU  (N=36)  Pilot | Monitoring (PMDC-R) (FR&MR) | Overall: p<.001 | Overall: maintenance |  |  |  |  |  |  |
|  |  |  | Diabetes conflict (DFCS) (FR&MR&CR) | FR&MR: p<.05  CR: N.S. | FR&MR: maintenance,  CR: N.S. |  |  |  |  |  |  |
|  |  |  | Helplessness (PHQ) (FR&MR) |  |  | Overall: p<.001 | Overall: maintenance |  |  |  |  |
|  |  |  | Diabetes care (SCI) (FR&MR&CR) |  |  |  |  |  |  | Overall: p<.001 | Overall: maintenance |
|  |  |  | BGM frequency (OM) |  |  |  |  |  |  | N.S. | N.S. |
|  |  |  | BG daily average (OM) |  |  |  |  |  |  | Overall: p<.03 | Overall: maintenance |
| Jaser 2018 | Communication and coping,  CAU | RM-ANOVA, group*time  Pre, post  (N=30)  Pilot | Parenting style (APQ) (PR) – *positive parenting* | N.S. |  |  |  |  |  |  |  |
|  |  |  | Diabetes conflict (DFCS) (PR) | p<.05, ES=1.42 |  |  |  |  |  |  |  |
|  |  |  | Diabetes conflict (DFCS) (CR) | p<.05, ES=1.46 |  |  |  |  |  |  |  |
|  |  |  | Diabetes distress (DDS-P) (PR) |  |  | p<.05, ES=1.43 |  |  |  |  |  |
|  |  |  | Depressive symptoms (PHQ-9) (PR) |  |  | N.S. |  |  |  |  |  |
|  |  |  | Anxiety symptoms (STAI) (PR) |  |  | N.S. |  |  |  |  |  |
|  |  |  | Depressive symptoms (CDI) (CR) |  |  |  |  | N.S. |  |  |  |
|  |  |  | Quality of life (PedsQL Diabetes) (CR) |  |  |  |  | p<.05, ES=1.68 |  |  |  |
|  |  |  | Diabetes self-care (SCI) (CR) |  |  |  |  |  |  | N.S. |  |
|  |  |  | HbA1c (OM) |  |  |  |  |  |  | N.S. |  |
| Kawamura 2012 *(conf. abstr.)* | MI training,  waitlist control | NR;  Pre, 6 mo. FU, 12 mo. FU  (N=NR) | Depressive symptoms (CDI) (CR) |  |  |  |  |  | I: NR  C: p<.05 |  |  |
|  |  |  | HbA1c (NR) |  |  |  |  |  |  | I: p<.01  C: N.S. | I: NR  C: NR |
| Supplementary Table 1 – Extracted data of included studies on post and first follow up measures *(continued – 14)* | | | | | | | | | | | |
| Reports (first author, year) | Intervention, control | Reported effects (analyses, time-points, N) | Outcome (measure) (modality) - *(subscale)* | Parenting/  Family dynamics | | Parent | | Child | | Diabetes | |
|  |  |  |  | Post | FU | Post | FU | Post | FU | Post | FU |
| Liberman 2017 *(conf. abstr.)* | Parental authoritativeness,  educational control (EC), CAU | NR;  Pre, 12 mo. FU  (N=33) | HbA1c |  |  |  |  |  |  |  | I: p<.05  EC/CAU: both N.S. |
| **Contracting interventions** | | | | | | | | | | | |
| Hannon, 2018; Halper, 2022 | Family goal setting,  no control | ^$^ANOVA;  ^$$^linear mixed models  pre, post, 6 mo. FU  (N=33)  Pilot | Conflict (PEQPCCS) (PR) |  | N.S. ^$$^ |  |  |  |  |  |  |
|  |  |  | Diabetes distress (PAID) (PR&CR) |  |  |  | N.S. ^$$^ |  | N.S. ^$$^ |  |  |
|  |  |  | Quality of life (PedsQL Diabetes) (CR) – *5 subscales* |  |  |  |  |  | all N.S. ^$$^ |  |  |
|  |  |  | Sleepiness (CASQ) (CR) |  |  |  |  |  | N.S. ^$$^ |  |  |
|  |  |  | Diabetes self-care (ADQ) (CR) |  |  |  |  |  |  |  | p=.002^$$^ |
|  |  |  | HbA1c (OM) |  |  |  |  |  |  | N.S. ^$^ | N.S. ^$^ |
|  |  |  | BGM frequency (OM) |  |  |  |  |  |  | N.S.^$^ | N.S. ^$^ |
| Carroll, 2011 | Behavioral contracting,  no control | Regression;  Pre, post;  (N=10)  Pilot | Parenting behavior (CPBDS) (CR) | N.S. |  |  |  |  |  |  |  |
|  |  |  | Miscarried helping (HHI) (CR) | NR |  |  |  |  |  |  |  |
|  |  |  | Quality of life (PedsQL – Diabetes) (CR) |  |  |  |  | N.S. |  |  |  |
|  |  |  | Diabetes self-care (DSMP) (I - respondent NR) |  |  |  |  |  |  | p<.01 |  |
|  |  |  | HbA1c (OM) |  |  |  |  |  |  | p<.04 |  |
| Stanger, 2013 | Contingency management,  no control | Mixed models, d_rm_ = RM effect size;  ^$^p adjusted (Tukey);  Pre, post, (~ 6.5 mo. FU for HbA1c)  (N=17)  Pilot | Diabetes self-care (SCI) (PR) |  |  |  |  |  |  | p=.0002,  d_rm_ = -1.21 |  |
|  |  |  | Diabetes self-care (SCI) (CR) |  |  |  |  |  |  | p=.041,  d_rm_ = -0.58 |  |
|  |  |  | BGM frequency (OM) |  |  |  |  |  |  | p=.002,  d_rm_ = -1.00 |  |
|  |  |  | HbA1c (OM) |  |  |  |  |  |  | p<.001^$^, d_rm_=1.25 | p<.004^$^, d_rm_=0.95 |
| Epstein 1981 | Behavioral program,  multiple baseline | Analysis not conducted;  (N=19) | % negative urine glucose tests |  |  |  |  |  |  | NR | NR |
|  |  |  | HbA1c |  |  |  |  |  |  | NR | NR |

| Supplementary Table 1 – Extracted data of included studies on post and first follow up measures *(continued – 15)* | | | | | | | | | | | |
| --- | --- | --- | --- | --- | --- | --- | --- | --- | --- | --- | --- |
| Reports (first author, year) | Intervention, control | Reported effects (analyses, time-points, N) | Outcome (measure) (modality) - *(subscale)* | Parenting/  Family dynamics | | Parent | | Child | | Diabetes | |
|  |  |  |  | Post | FU | Post | FU | Post | FU | Post | FU |
| **Digital interventions** | | | | | | | | | | | |
| Hilliard 2020 | T1 Doing Well,  CAU | Independent t-tests on mean change  Pre, post;  (N=80)  Pilot feasibility | Miscarried helping (HHI) (PR&CR) | both N.S. |  |  |  |  |  |  |  |
|  |  |  | Diabetes conflict (DFCS) (PR&CR) | both N.S. |  |  |  |  |  |  |  |
|  |  |  | Relationship quality (PYRI, adapted) (PR+CR) | N.S. |  |  |  |  |  |  |  |
|  |  |  | Family impact (PedsQL Family Impact) (PR) |  |  | N.S. |  |  |  |  |  |
|  |  |  | Diabetes distress (PAID) (PR) |  |  | N.S. |  |  |  |  |  |
|  |  |  | Diabetes strengths (DSTAR) (CR) |  |  |  |  | N.S. |  |  |  |
|  |  |  | Quality of life (My-Q) (CR) |  |  |  |  | N.S. |  |  |  |
|  |  |  | Diabetes distress (PAID) (CR) |  |  |  |  | N.S. |  |  |  |
|  |  |  | Diabetes self-care (DSMP) (I - PR) |  |  |  |  |  |  | N.S. |  |
|  |  |  | Diabetes self-care (SCI) (CR) |  |  |  |  |  |  | N.S. |  |
|  |  |  | HbA1c (OM) |  |  |  |  |  |  | N.S. |  |
| Whittemore 2020 | T1 Teamwork,  waitlist control | RM regression, group*time;  pre, post, 6 mo. FU  (N=162) | Autonomy support (DSPSA) (PR) | NR | N.S. |  |  |  |  |  |  |
|  |  |  | Diabetes conflict (DFCS) (PR) | NR | N.S. |  |  |  |  |  |  |
|  |  |  | Parenting stress (PIP) (PR) - *frequency* |  |  | NR | b=-1.196, p=.04 |  |  |  |  |
|  |  |  | Parenting stress (PIP) (PR) - *difficulty* |  |  | NR | b=-1.361, p=.05 |  |  |  |  |
|  |  |  | General stress (PSS) (PR) |  |  | NR | N.S. |  |  |  |  |
|  |  |  | Anxiety (STAI) (PR) |  |  | NR | N.S. |  |  |  |  |
|  |  |  | Depressive symptoms (CES-D) (PR) |  |  | NR | N.S. |  |  |  |  |
|  |  |  | HbA1c (PR) |  |  |  |  |  |  | NR | N.S. |
| **Young children interventions** | | | | | | | | | | | |
| Tully 2018 | TOTs,  no control | Generalized estimation equation + estimate (GEE)  Pre, post ~6 mo. FU  (N=10)  Feasibility | Parenting distress (PIP) (PR) - *frequency* |  |  | p=.007, GEE=-11.44 | NR |  |  |  |  |
|  |  |  | Parenting distress (PIP) (PR) - *difficulty* |  |  | p<.001, GEE=-18.11 | NR |  |  |  |  |
|  |  |  | Depressive symptoms (CES-D) (PR) |  |  | p=.034, GEE=-5.78 | NR |  |  |  |  |
|  |  |  | Negative affect feeding (BPFAS) (PR) |  |  | p=.014, GEE=-1.56 | NR |  |  |  |  |
|  |  |  | Child feeding behavior (BPFAS) (PR) – *frequency* |  |  |  |  | N.S. | N.S. |  |  |
|  |  |  | Child feeding behavior (BPFAS) (PR) – *problem* |  |  |  |  | N.S. | p<.05, GEE= -2.96 |  |  |
|  |  |  | HbA1c (OM) |  |  |  |  |  |  | N.S. | N.S. |
|  |  |  | TIR (OM) |  |  |  |  |  |  | NA | NA |
|  |  |  | BG level (OM) |  |  |  |  |  |  | p=.016 | N.S. |

| Supplementary Table 1 – Extracted data of included studies on post and first follow up measures *(continued – 16)* | | | | | | | | | | | |
| --- | --- | --- | --- | --- | --- | --- | --- | --- | --- | --- | --- |
| Reports (first author, year) | Intervention, control | Reported effects (analyses, time-points, N) | Outcome (measure) (modality) - *(subscale)* | Parenting/  Family dynamics | | Parent | | Child | | Diabetes | |
|  |  |  |  | Post | FU | Post | FU | Post | FU | Post | FU |
| Mackey 2022 | TOT,  CAU | Paired t tests per group  Pre, post, 6 mo. FU;  (N=36)  Pilot | Depressive symptoms (CES-D) (PR) |  |  | NR | I: p<.05  C: N.S. |  |  |  |  |
|  |  |  | Negative affect feeding (BPFAS) (PR) – *parent problem* |  |  | NR | p<.05  C: N.S. |  |  |  |  |
|  |  |  | Child eating behavior (BPFAS) (PR) –*problems* |  |  |  |  | NR | I: p<.05  C: N.S. |  |  |
|  |  |  | HbA1c (OM) |  |  |  |  |  |  | NR | I: N.S.  C: N.S. |
|  |  |  | Time-in-range (OM) |  |  |  |  |  |  | NR | I: p<.05  C: N.S. |
| Patton 2014 | BEST MEALS,  no control | Paired t-tests  Pre, post  (N=9)  Pilot | Mean daily glucose level (OM) |  |  |  |  |  |  | p<.001, d=0.60 |  |
| Patton 2020 | REDCHiP,  waitlist control | RM-ANOVA group*time  pre, post, 5,5 mo. FU  (N=43)  Pilot | Fear of hypoglycemia (HFS-PYC) (PR) |  |  | p=.04, η_p_ =.117 |  |  |  |  |  |
|  |  |  | Parenting stress (PIP) (PR) - *frequency* |  |  | N.S. |  |  |  |  |  |
|  |  |  | Parenting stress (PIP) (PR) - *difficulty* |  |  | N.S. |  |  |  |  |  |
|  |  |  | Diabetes distress (PAID) (PR) |  |  | N.S. |  |  |  |  |  |
|  |  |  | HbA1c (OM) |  |  |  |  |  |  | NR |  |
| Reported results are from unadjusted analyses, unless otherwise specified. Results presented in *italics* represent effects that do not favor the intervention group; results presented in the middle of two cells indicate that no post-hoc contrasts between time-points were reported by authors. Timing of follow-up measure is relative to baseline.  For all findings, p-values and effect sizes were extracted, if available. Missing p-values were labeled as 'not reported' (NR); effect sizes were extracted when available, with 'not reported' omitted to maintain readability.  FU= follow up time (reference pre=intervention); PR= parent report; CR= child report; FR = father report; MR = mother report; CLR = clinician rated; I = interview; OR = observer reported; OM = objectively measured; mean PR+CR = averaged PR and CR; PR or CR = one of aforementioned reported (e.g. depending on age, completeness data etc.); PR&CR = both parent and child  I = intervention; CAU = care as usual; RM-ANOVA = repeated measures ANOVA; mo. = month; FU = follow-up; sign = significant; N.S. = not significant; NR = not reported; NA = not applicable; BG = blood glucose; BGM = blood glucose monitoring; DKA = diabetic ketoacidosis; TIR = time-in-range; CwD = child with diabetes; ES = effect size;  Annotations between brackets illustrate direction of interaction effects, i.e. increase (+), stabilization (=) or decrease (-) for the intervention (I) or control (C) group. ^#^ indicates these directions are based on visual inspection and did no undergo statistical testing.  PARQ = Parent-Adolescent Relationship Questionnaire; IC = Issues Checklist; DRCS = Diabetes Responsibility and Conflict Scale; IBC = Interaction Behavior Code; TADS = Teen Adjustment to Diabetes Scale; SCI = Self Care Inventory; DSMP = Diabetes Self-Management Profile; CBQ = Conflict Behavior Questionnaire; DFBC = Diabetes Family Behavior Checklist; AIS = Adjustment to Illness Scale; CBCL = Child Behavior Check List; DMQ = Diabetes Mismanagement Questionnaire; HHI = Helping for Health Inventory; DFCS = Diabetes Family Conflict Scale; CDI = Children's Depression Inventory; DFBS = Diabetes Family Behavior Scale; DFRQ = Diabetes Family Responsibility Questionnaire; CPRS = Child–Parent Relationship Scale; DMS = Diabetes Management Scale; GHb = total glycohemoglobin; DSQ = Diabetes Stress Questionnaire; DQOL = Diabetes Quality of Life Scale for Youth; PMDC(-R) = Parental Monitoring of Diabetes Care Scale (-Revised); RRR= Rollnick's Readiness Ruler; PedsQL = Paediatric Quality of Life Inventory; PAID = Problem Areas in Diabetes; BGM-HIS = Blood Glucose Monitoring/Health Information Survey; CPI = Collaborative Parent Involvement scale; PMDCS = Parental Monitoring of Diabetes Care scale; PIS = Parental Involvement Scale; FES = Family Environment Scale; SEDSM = Self-Efficacy for Diabetes Self-Management scale; DBRS = Diabetes Behavior Rating Scale; RCBRS = Readiness to Change the Balance or Responsibility Scale; BSI-18 = Brief Symptom Inventory; BASC-2 = Behavioral Assessment Scales for Children; FACES II = Family Adaptability and Cohesion Scale; ICIDDM = Issues in Coping with IDDM; CES-D = Center for Epidemiologic Depression Scale; PDQOL = Parents Diabetes Quality of Life questionnaire; SEDS = Self-Efficacy for Diabetes Scale; PHQ = Parental Helplessness Questionnaire; PS = Parenting Scale; QEB = Questions to Education Behaviour; DASS = Depression-Anxiety-Stress Scale; SDQ = Strengths and Difficulties Questionnaire; PSOC = Parenting Sense of Competence scale; APQ = Alabama Parenting Questionnaire; DDS-P = Diabetes Distress Scale; PHQ-9 = Patient Health Questionnaire; STAI = State Trait Anxiety Inventory; PIP = Paediatric Inventory for Parents; ECBI = Eyberg Child Behavior Inventory; PPC = Parent Problem Checklist; RQI = Relationship Quality Index; PARI = Parental Attitude Research Instrument; CBAQ = Conflict Behaviour Assessment Questionnaire; GHQ-12 = General Health Questionnaire; HFS-PYC = Hypoglycemia Fear Survey for Parents of Young Children; DFI-S = Diabetes Family Impact Scale; WHO-5 = World Health Organization Well-Being Index ;IOS = Inclusion of Others in the Self scale; MIE = Measure of Intimate Events; MOS = Mealtime Observation Schedule; CAPES = Child Adjustment and Parent Efficacy Scale; DBC = Diabetes Behavior Checklist; PSI-SF = Parenting Stress Index/Short Form; PECIS = Parent Experience of Child Illness Scale; WEMWBS = Warwick Edinburgh Mental Wellbeing Scale; DEPS-R = Diabetes Eating Problem Survey – Revised; CEBQ = Child Eating Behaviour Questionnaire; PEQPCCS = Parental Environment Questionnaire Parent-Child Conflict Scale; CASQ = Cleveland Adolescent Sleepiness Questionnaire; ADQ = Adherence in Diabetes Questionnaire; CPBDS = Cornell Parent Behavior Description Scale; DSPSA = Diabetes-Specific Parental Support for Autonomy Scale; PSS = Perceived Stress Scale; PYRI = Parent-Youth Relationship Index; DSTAR = Diabetes Strengths and Resilience measure; BPFAS = Behavioral Pediatrics Feeding Assessment Scale;  **Alt text:** Table showing findings (p-values, effect sizes) of post- and first follow-up outcomes (including measure, modality and subscale) for parenting/family dynamics, parent outcomes, child outcomes, and diabetes outcomes. Reports are grouped by eight different intervention types and multiple reports of the same study are collated. | | | | | | | | | | | |
