# Supplementary material for: Parenting interventions for parents of children with type 1 diabetes—a systematic review
Source: J Pediatr Psychol. 2025 Sep 22;50(12):1115–38. doi: 10.1093/jpepsy/jsaf078 (PMC12755088; doi:10.1093/jpepsy/jsaf078)
Supplement: jsaf078_Supplementary_Data [file jsaf078_supplementary_data.zip › jsaf078_Supplementary_Data/jpepsy-2024-0314-File012_final.docx]

| Supplementary Table S2 - Additional analyses of included studies | | | | |
| --- | --- | --- | --- | --- |
| **Reports (first author, year)** | **Intervention, control** | **Additional analyses** | **Outcomes** | **Findings** |
| Wysocki, 1999;  Wysocki, 2000;  Wysocki, 2001 | Behavioral family systems therapy (BFST),  educational control (EC), CAU | Moderation | Adjustment to T1D (TADS) (mean PR+CR) | **Mod** group*gender and group*age *gender. For boys, better adjustment after BFST and worse after EC, for girls vice versa. |
|  |  |  | HbA1c (OM) | **Mod** group*age: older children in BFST increase vs. decrease in younger children.  group*age*gender: among BFST participants, older girls demonstrated increase in HbA1c, vs. big decreases for younger girls, and moderate decreases for both younger and older boys. |
| Wysocki, 2006; Wysocki, 2007; Wysocki, 2008; | Behavioral family systems therapy - diabetes (BFST-D),  educational control (EC), CAU | Moderation | Conflict (DRCS) (mean PR+CR) | **Mod** (post) group*time*baseline HbA1c (p<.04): greater reduction in BFST-D, but only for those with baseline HbA1c ≥ 9.0%. Best described as “BFST-D attenuating the increases of conflict that would be expected and occurred in educational control and care-as-usual”. |
|  |  |  | Diabetes self-care (DSMP) (I) (mean PR+CR) | **Mod** (post) group*time*baseline HbA1c (p<.03): BFST-D showed greater reduction in DSMP scores than EC and CAU within both baseline HbA1c ranges; and especially among those with baseline HbA1c > 9% |
|  |  |  | HbA1c (OM) | **Mod** (post) group*time*baseline HbA1c (p<.05): BFST-D was superior to CAU, but not EC, among those with baseline HbA1c > 9.0% |
| Harris 2015,  Riley 2015, Duke 2016 | Behavioral family systems therapy - diabetes (BFST-D)  no control | Online vs. face-to-face;  Mediation  SG: elevated baseline child depression | all outcomes | Online vs. face-to-face N.S. for all outcomes |
|  |  |  | Miscarried helping (HHI) (PR) | **Med:** relation with illness adjustment mediated by family conflict |
|  |  |  | Child depressive symptoms (CDI) (CR) | **SG**: replicated overall effects (p<.001 for post and FU together). |
| Ellis 2005a; Ellis 2005b; Ellis 2007a; Ellis 2007b;  Naar-King 2007 | Multisystemic treatment (MST),  CAU | Moderation and mediation | Caregiver support (DFBC) (PR+CR) - *supportive* | Main effect on secondary caregiver in 2 parent household (p<.05)  **Mod** (post) group*family composition (p≤.05): MST improved primary caregiver support for 2-parent (p=.01) but not for single parent families (p=.18); CAU N.S. |
|  |  |  | Family relationships (FES) (PR+CR) – *Family Relationship Index* | **Mod** (post) group*family composition (p<.05): however, simple effects single parent and two-parent both N.S. |
|  |  |  | Parental overestimation of child responsibilities (DFRQ, adapted) (PR+CR combined) | **Mod:** No moderation of demographic variables |
|  |  |  | Diabetes self- care – BGM frequency (OM) | **Mod**:  Post & FU (2007a): treatment * time * family composition (p<.05): in 2 parent families initial post- effects (p<.01, ES=0.67) were maintained at follow up (p<.001, ES=0.53); in 1 parent families initial effects (p<.012) were not maintained (N.S.);  Post (2007b): treatment * family composition N.S.  **Med:**  Family relations did not mediate effect of MST on BGT; MST had a direct effect on BGT.  Stress did not mediate effect of MST on diabetes self-care. |
|  |  |  | DKA admission rate (relative DKA drop in MST /relative DKA drop in control) (OM) | **Mod:** No moderation of demographic variables |
|  |  |  | HbA1c (OM) | **Mod** (post, 2007b): treatment*family composition N.S. (or any other demographic; 2007a)  **Med** (post, 2007b): family relations did not mediate effect of MST on HbA1c.  Stress did not mediate effect of MST on HbA1c.  BGM frequency mediates relation of MST on HbA1c in single but not two-parent families (p=.04) |

| Supplementary Table 2 - Additional analyses of included studies (*continued – 2*) | | | | |
| --- | --- | --- | --- | --- |
| **Reports (first author, year)** | **Intervention, control** | **Additional analyses** | **Outcomes** | **Findings** |
| Svoren 2003 | Family psychoeducation (PE) + care ambassador (CA),  CA only/CAU (combined) | SG: A1c ≥ 8.7% | HbA1c – mean past 24 months (OM) | **SG**: p=.01 for I vs. CA-only/CAU group. |
| Katz 2014 | Family psychoeducation (PE) + care ambassador (CA),  CA only, CAU | SG: A1c≥8% (N = 56); pre, post (bivariate analyses) | Parent T1D responsibilities (DFRQ) (mean PR+CR) Diabetes conflict (DFCS) (PR&CR)  Quality of life (PedsQL-Generic) (PR&CR) | all N.S. |
|  |  |  | HbA1c (OM) | N.S. – “After 2 years, the adjusted A1C in participants with initial A1c ≥8.0% was comparable.” |
| Nansel 2012, Nansel 2015, Gee 2017,  Temmen, 2022  Lu, 2023 | WE-CAN,  CAU | Markov model^$^;  Moderation  Separate analyses per age group | Parental involvement and conflict (DFRQ and DFCS) (PR&CR) | Lu, 2023: “Families in the intervention arm were more likely to stay in the Harmonious parent–child relation state [ high involvement, low conflict] and less likely to move from Harmonious to Indifferent [low involvement, low conflict]. Intervention families were also less likely to stay in Discordant [high involvement, high conflict] and more likely to move from Discordant to Harmonious state.”^$^ |
|  |  |  | HbA1c (OM) | Separate analyses per age group: effects on HbA1c only among older adolescents (12-14years) (p=.009) resulting from increase in CAU condition and minor increase in intervention condition; no effects in younger group (9-11 years) (N.S.).  **Mod** treatment*income: no moderation of income.  Intervention effect did not differ across parenting classes (Harmonious/Discordant/ Indifferent) |
| Ellis 2024, Knauft 2024 | 3Ms | Moderation (within intervention group) | HbA1c | **Mod:** Within the intervention group, most prominent decreases in HbA1c were found in the high depressive symptom subgroup at 6 month after completion (“18 month FU” in paper) (p=.002).  **Mod**; group*time*baseline diabetes distress N.S. Within the intervention group, decreases in HbA1c were found only for those with high baseline diabetes distress, not for low baseline diabetes distress or in the standard care group. |
| Rothman Kabir, 2022 | New Authority (NA) training,  no control | Implementation of learned skills | Implementation of NA skills (I) | The skills ‘escalation prevention’, ‘family dining together’ and ‘diabetes meetings’ were applied by more than 60% of the families. At follow-up (T4), families reported continued use of ‘diabetes meetings’ and ‘family dining together. |

| Supplementary Table 2 - Additional analyses of included studies (*continued – 3*) | | | | | |
| --- | --- | --- | --- | --- | --- |
| **Reports (first author, year)** | **Intervention, control** | **Additional analyses** | **Outcomes** | **Findings** | |
| Westrupp 2014 | Triple P,  CAU | SG:  pre-existing child behavior problems; | Ineffective discipline strategies (PS) (PR) - *Total* | SG 3mo. N.S.; SG 12mo. p=.049 | |
|  |  |  | Conflict over parenting (PPC) (PR) | SG 3mo. p=.007, ES=0.96; SG 12mo. N.S. | |
|  |  |  | Diabetes conflict (DFCS) (PR) | SG 3mo. p=.034, ES=0.82; SG 12mo. N.S. | |
|  |  |  | Marital quality (RQI) (PR) | SG 3mo. N.S.; SG 12mo. N.S. | |
|  |  |  | Child behavior (BASC-2) (PR) – *parent rating scale -* *internalize* | SG 3mo. p=.036, ES=0.77; SG 12mo. N.S. | |
|  |  |  | Child behavior (BASC-2) (PR) - *parent rating scale -externalize* | SG 3mo. p=.038, ES=0.90; SG 12mo. N.S. | |
|  |  |  | Disruptive behavior (ECBI) (PR) - *intensity* | SG 3mo. p=.024, ES=0.88; SG 12mo. N.S. | |
|  |  |  | Disruptive behavior (ECBI) (PR) - *number* | SG 3mo. p=.019, ES=0.63; SG 12mo. N.S. | |
|  |  |  | Depressive symptoms (DASS) (PR) | SG 3mo. p=.019, ES=0.63; SG 12mo. N.S. | |
|  |  |  | Anxiety symptoms (DASS) (PR) | SG 3mo. p=.031, ES=0.68; SG 12mo. p=.020, ES=0.83 | |
|  |  |  | Stress symptoms (DASS) (PR) | SG 3mo. p=.022, ES=0.72; SG 12mo. p=.006, ES=1.03 | |
|  |  |  | View parenting role (PSOC) (PR) - *satisfaction* | SG 3mo. p=.049, ES=0.38; SG 12mo. N.S. | |
|  |  |  | View parenting role (PSOC) (PR) – *self-efficacy* | *SG 3mo. p=.018, ES=1.00*; SG 12mo. N.S. | |
|  |  |  | HbA1c (OM) | SG 3mo. N.S.; SG 12mo. N.S. | |
| Mitchell, 2022 | Healthy Living Triple P,  CAU | Effects on secondary caregiver; | Self-efficacy (DBC) (PR) - *Confidence* | p=.008, b=14.78 (C+, I=) | |
|  |  |  | Adjustment – parent (PECIS) (PR) – *4 subscales* | *Emotional resources* p=.041, b=0.23 (I=, C-).  Others N.S. | |
|  |  |  | Quality of life – child (PedsQL Generic) (PR) | p=.023, b=7.32 (I=, C=) | |
| Patton 2014 | BEST MEALS,  no control | Effect sizes without significance test | Parent feeding behavior (DINE) (OR) – *direct requests to eat* | d=0.77 (pre>post) | |
|  |  |  | Parent feeding behavior (DINE) (OR) – *indirect requests to eat* | d=0.61 (pre>post) | |
|  |  |  | Parent feeding behavior (DINE) (OR) – *parent talk* | d=0.56 (pre>post) | |
|  |  |  | Parent feeding behavior (DINE) (OR) – *coax* | d=0.79 (pre>post) | |
|  |  |  | Parent feeding behavior (DINE) (OR) – *physical prompts* | d=0.31 (pre>post) | |
|  |  |  | Parent feeding behavior (DINE) (OR) – *reinforcement* | d=-0.98 (pre<post) | |
|  |  |  | Parent feeding behavior (DINE) (OR) – *feed* | d=0.63 (pre>post) | |
|  |  |  | Child eating (DINE) (OR) - *bites* | d=0.63 (pre>post) | |
|  |  |  | Child eating (DINE) (OR) - *sips* | d=0.32 (pre>post) | |
|  |  |  | Child eating (DINE) (OR) – *compliance to requests* | d=1.13 (pre>post) | |
|  |  |  | Child eating (DINE) (OR) – *noncompliance to requests* | d=0.50 (pre>post) | |
|  |  |  | Child eating (DINE) (OR) – *child talk* | d=0.88 (pre>post) | |
|  |  |  | Child eating (DINE) (OR) – *requests for food* | d=-0.30 (pre<post) | |
|  |  |  | Child eating (DINE) (OR) - *play* | d=-0.64 (pre<post) | |
|  |  |  | Child eating (DINE) (OR) – *away from table* | d=-0.23 (pre<post) | |
|  |  |  | Child eating (DINE) (OR) - *refuse* | d=0.35 (pre>post) | |
| Patton 2020 | REDCHiP,  waitlist control | t-test pre-FU (5.5 mo.) | Fear of hypoglycemia (HFS-PYC) (PR) | I: p<.001; WC=NA |  |
|  |  |  | Parenting stress (PIP) (PR) - *frequency* | I: p=.001; WC=NA |  |
|  |  |  | Parenting stress (PIP) (PR) - *difficulty* | I: p=.002; WC=NA |  |
|  |  |  | Diabetes distress (PAID) (PR) | I: p=.009; WC=NA |  |
| Results presented in *italics* represent effects that do not favor the intervention group.  SG = subgroup analysis; med = mediation; mod = moderation; I = intervention; CAU = care-as-usual; WC = waitlist control; N.S. = not significant; BGM = blood glucose monitoring; mo. = months; DKA = diabetic ketoacidosis; NA = not applicable  FU= follow up time (reference pre-intervention); PR= parent report; CR= child report; I = interview; OR = observer reported; OM = objectively measured; mean PR+CR = averaged PR and CR; PR&CR = both parent and child  Annotations between brackets illustrate direction of interaction effects, i.e. increase (+), stabilization (=) or decrease (-) for the intervention (I) or control (C) group.  TADS = Teen Adjustment to Diabetes Scale; DRCS = Diabetes Responsibility and Conflict Scale; DSMP = Diabetes Self-Management Profile; HHI = Helping for Health Inventory; CDI = Children's Depression Inventory; DFBC = Diabetes Family Behavior Checklist; FES = Family Environment Scale; DFRQ = Diabetes Family Responsibility Questionnaire; DFCS = Diabetes Family Conflict Scale; PedsQL = Paediatric Quality of Life Inventory; PS = Parenting Scale; PPC = Parent Problem Checklist; RQI = Relationship Quality Index; BASC-2 = Behavioral Assessment Scales for Children; ECBI = Eyberg Child Behavior Inventory; DASS = Depression-Anxiety-Stress Scale; PSOC = Parenting Sense of Competence scale; DBC = Diabetes Behavior Checklist; PECIS = Parent Experience of Child Illness Scale; DINE = Dyadic Interaction Nomenclature for Eating; HFS-PYC = Hypoglycemia Fear Survey for Parents of Young Children; PIP = Paediatric Inventory for Parents; PAID = Problem Areas in Diabetes  **Alt text:** Table showing findings of additional analyses on post and first follow up measures of included reports (controlled and uncontrolled studies). Extracted additional analyses include moderation analyses, mediation analyses, subgroup analyses, effects on secondary caregivers, and findings that did not undergo statistical testing due to small sample sizes. | | | | | |
